# Advances in Flavonoid Research: Sources, Biological Activities, and Developmental Prospectives

**DOI:** 10.3390/cimb46040181

**Published:** 2024-03-26

**Authors:** Baocheng Hao, Zhen Yang, Haoyu Liu, Yu Liu, Shengyi Wang

**Affiliations:** Key Laboratory of New Animal Drug Project, Gansu Province, Key Laboratory of Veterinary Pharmaceutical Development, Ministry of Agriculture and Rural Affairs, Lanzhou Institute of Husbandry and Pharmaceutical Sciences of Chinese Academy of Agriculture Sciences, Lanzhou 730050, China; haobaocheng@caas.cn (B.H.); lzmy_yz@163.com (Z.Y.); lhy000503@163.com (H.L.); liuyu8108@163.com (Y.L.)

**Keywords:** flavonoids, biosynthesis, bioactivity, mechanism, drug delivery systems

## Abstract

At present, the occurrence of a large number of infectious and non-communicable diseases poses a serious threat to human health as well as to drug development for the treatment of these diseases. One of the most significant challenges is finding new drug candidates that are therapeutically effective and have few or no side effects. In this respect, the active compounds in medicinal plants, especially flavonoids, are potentially useful compounds with a wide range of pharmacological activities. They are naturally present in nature and valuable in the treatment of many infectious and non-communicable diseases. Flavonoids are divided into fourteen categories and are mainly derived from plant extraction, chemical synthesis and structural modification, and biosynthesis. The structural modification of flavonoids is an important way to discover new drugs, but biosynthesis is currently considered the most promising research direction with the potential to revolutionize the new production pipeline in the synthesis of flavonoids. However, relevant problems such as metabolic pathway analyses and cell synthesis protocols for flavonoids need to be addressed on an urgent basis. In the present review, new research techniques for assessing the biological activities of flavonoids and the mechanisms of their biological activities are elucidated and their modes of interaction with other drugs are described. Moreover, novel drug delivery systems, such as nanoparticles, bioparticles, colloidals, etc., are gradually becoming new means of addressing the issues of poor hydrophilicity, lipophilicity, poor chemical stability, and low bioavailability of flavonoids. The present review summarizes the latest research progress on flavonoids, existing problems with their therapeutic efficacy, and how these issues can be solved with the research on flavonoids.

## 1. Introduction

At present, the occurrence of a large number of infectious and non-communicable diseases poses a serious threat to human health; for example, the invasion and transmission of COVID-19 have been a nightmare for humanity globally since 2020. As a result, the research and development cycle for therapeutic drugs for these diseases faces enormous challenges.

One of the most significant challenges is finding new drug candidates which are therapeutically effective and have few or no side effects. In this aspect, the active compounds in medicinal plants, especially flavonoids, are potentially useful compounds with a wide range of pharmacological activities. They are naturally present in nature and valuable in the treatment of many infectious and non-communicable diseases. 

**Figure 1:**
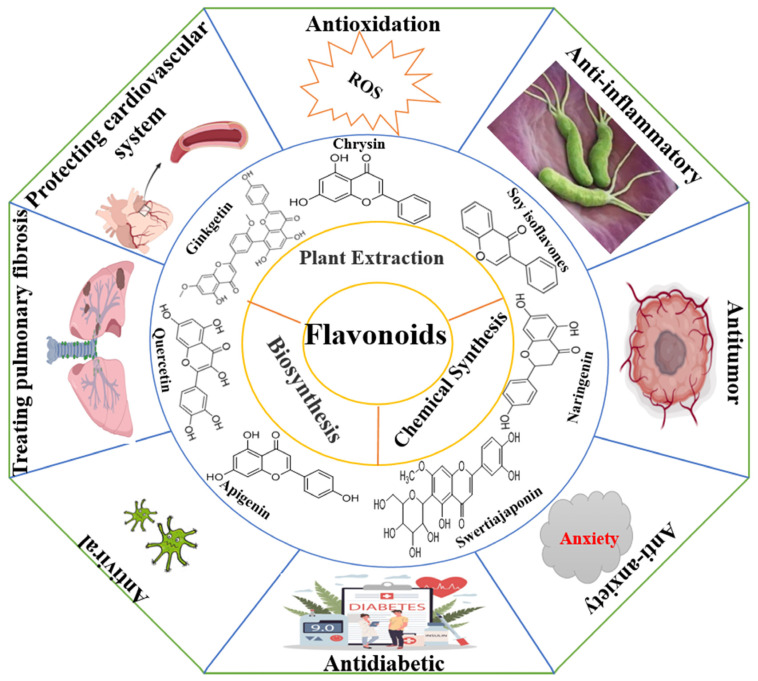
Flavonoids have a wide range of biological activities.

Flavonoids are widely distributed in the roots, stems, leaves, flowers, and fruits of higher plants such as *Rutaceae*, *Lamiaceae*, *Leguminosae*, *Apiaceae*, *Ginkgoaceae*, and *Asteraceae* and are one of the secondary metabolites produced by long-term natural selection [1,2]. There are a wide variety of flavonoids, and since the discovery of the first flavonoid, chrysin (**5**,**7**-dihydroxyflavone), in 1814, more than 10,000 compounds have been isolated and identified [3]. Flavonoids are synthesized via the phenylpropane pathway and exist in nature as glycosidic ligands, glycosides, and methylated derivatives. Flavonoids comprise an important class of information molecules involved in the regulation of physiological functions and play a vital role in all aspects of the plant growth process. In 1990, researchers found that the biological activity of flavonoid compounds was similar to that of Vitamin C and named after Vitamin “P”, “pycnogenol”, a total flavonoid extracted from *Pinus pinaster*, is now recognized by the Food and Drug Administration (FDA) as an edible flavonoid nutraceutical. Further research has found that the antioxidant effect of pycnogenol is much stronger than that of Vitamin C and Vitamin E, especially with respect to its healthcare benefits for the skin and blood vessels. Additionally, it can reach the brain through the blood–brain barrier to prevent and control diseases of the central nervous system, so it has received widespread attention. In recent decades, the accumulated evidence has shown that flavonoid compounds have a variety of biological activities, such as anti-tumor, antibacterial, antioxidant, anti-inflammatory, cardiovascular protection, anti-arrhythmic, anti-viral, hormone-like, and anti-allergic, and can modulate immune function (Figure 1) [4,5,6,7,8,9]. With concern about health issues growing, the demand for flavonoids in the medical and healthcare market continues to grow, and flavonoids remain a research hot spot among the many active small molecules in natural medicines. In this paper, the structural characteristics and classification, main sources, biological activities, pharmacological targets, mechanisms of action, and clinical synergies of flavonoids and the opportunities and challenges for flavonoid research are reviewed.

## 2. Structural Characteristics and Classification of Flavonoids from Natural Sources

**Figure 2:**
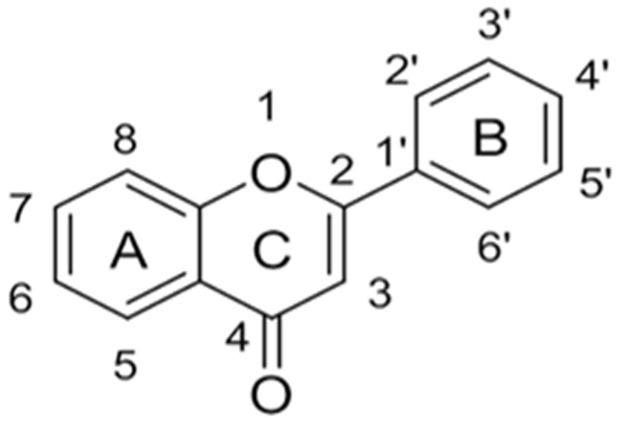
The “C6-C3-C6” mother core structure of flavonoids.

Originally, compounds with two aromatic rings, A and B, joined by a three-carbon chain were referred to as flavonoid compounds. The fundamental structure of a flavonoid compound is 2-phenyl chromone (Figure 2). Plants differ in the kinds and amounts of flavonoid chemicals they contain because of several factors that affect them, including species, growth stage, and growing environment. In addition, the structures of flavonoids are also diverse, including monomers, dimers, trimers, and tetramers. The number of novel flavonoid compounds continues to increase year by year with the depth of research. According to the degree of oxidation of the three-carbon chain, whether the three-carbon chain is ring-forming, and the position of the B-ring attached to the C-ring, among other distinguishing characteristics, flavonoids can be divided into the fourteen categories described below (Table 1) [3].

## 3. Sources of Flavonoids

### 3.1. Plant Extraction 

Natural flavonoids are polyphenols found in plants, which are the internal signaling molecules and intermediates or metabolites of plants. They are concentrated in angiosperms. For example, flavonoids are mostly found in plants such as *Lamiaceae*, *Acanthaceae*, *Gesneriaceae*, *Scrophulariaceae*, *Asteraceae*, etc. Flavonols are more widely distributed in dicotyledonous plants, dihydroflavonoids are widely dispensed in *Rosaceae*, *Rutaceae*, *Leguminosae*, *Ericaceae*, *Asteraceae* and *Zingiberaceae*, dihydro-flavonols are usually present in plants of the *Leguminosae*, and isoflavonoids present mainly in *Leguminosae*, *Papilionoideae,* and *Iridaceae*. In addition, flavonoids occur in a small number of gymnosperms, such as bioflavonoids existing in the plants of *Coniferopsida* and *Ginkgopsida*. Furthermore, flavonoids are also abundant in the edible pulp of vegetables and fruits, such as *Capsicum annuum* var. grossum, *Brassica oleracea*, *Allium cepa*, *Solanum lycopersicum*, *Citrus sinensis*, *Citrus limonum*, *Vitis vinifera*, *Citrus paradisi*, etc. (Table 2). 

Solvent extraction [10], ultrasonic extraction [11], microwave extraction, supercritical fluid extraction, gel permeation chromatography, ion-exchange chromatography, and other extraction methods are still the common methods for extracting flavonoids from plants. Furthermore, various enhanced and novel extraction techniques have also been documented. For instance, Biswas et al. optimized the ultrasound-assisted extraction (UAE) of total flavonoids from the leaves of *Corchorus olitorius* by applying response surface methodology (RSM), high-performance liquid chromatography (HPLC), and liquid chromatography–tandem mass spectrometry (LC-MS) analysis revealed that the extracts obtained by the optimized method contained isoquercetin and hypericin [12]. *Tamarindus indica* is a traditional Chinese medicine rich in flavonoids, triterpenes, and polyphenols as well as other active ingredients. Cui et al. extracted all of the flavonoids from *Tamarindus indica* using ultrasonography-assisted deep eutectic solvent (DES) extraction; the best extraction result was obtained using DES with **1**-proline and **1**,**2**-propylene glycol as the raw materials. The optimized extraction conditions were as follows: the water content of the solvent was 20%, ultrasonic power was 300 W, the extraction time was 40 min, the ratio of solid-to-liquid was 60 mg/mL, and the maximum yield of flavonoids was 26.54 mg/g [13]. In conclusion, there are numerous ways to extract flavonoids from plants; however, to obtain the best extraction effect, the most appropriate extraction method should be chosen based on the goals of the experiment, the traits of the plants, and the experimental setup.

**Table 2:** Part of flavonoid compounds extracted and isolated from plants.

Category	Main Chemical	Chemical Structure	Source of Plant		References
			Classes/Families	Species	
Flavonoid	Apigenin	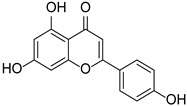	Lamiaceae, Acanthaceae, Gesneriaceae, Scrophulariaceae, Asteraceae, Verbenaceae, Rosaceae	*Apium graveolens*, *Coriandrum sativum*	[14,15]
	Baicalein	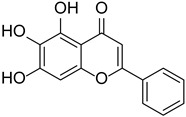		*Scutellaria*	[16]
	Nobiletin	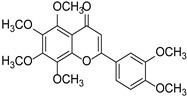		*Citrus*	[17]
	Chrysin	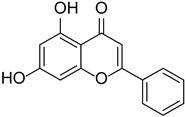		*Passiflora caerulea*	[18,19]
	Acacetin	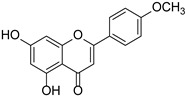		*Acacia farnesiana*, *Chrysanthemum*, *Robinia pseudoacacia*, *Buddleja officinalis*, *Chromolaene odorata*, *Cirsium japonicum*	[20]
	Oroxylin **A**	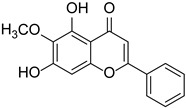		*Scutellaria baicalensis*	[21]
	Luteolin	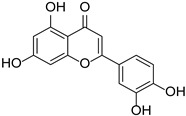		*Thymus mongolicus*, *Lonicera japonica*	[22,23]
	Eupatilin	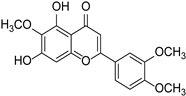		*Artemisia argyi*	[24]
	Eupatorin	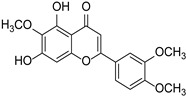		*Clerodendranthus spicatus*	[25]
	Vitexin	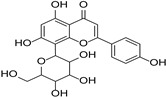		*Crataegus pinnatifida*	[26]
Flavonols	Fisetin	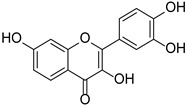	Rosaceae, Cucurbitaceae	*Fragaria* × *ananassa*, *Malus domestica*, *Cucumis sativus*	[27]
	Gossypin	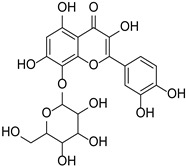		*Sphenodesme involucrata* var. paniculata	[28]
	Myricetin	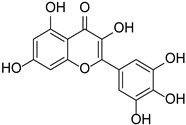		*Myrica esculenta*	[29]
	Quercetin	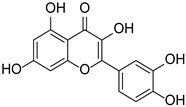		*Fagopyrum esculentum* rods and leaves, *Hippophae rhamnoides*, *Crataegus pinnatifida*, *Allium cepa*.	[30,31]
	Rutin	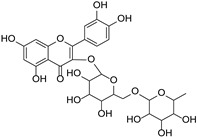		*Ruta graveolens*	[32]
Dihydroflavonoids	Naringenin	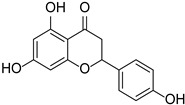	Rosaceae, Rutaceae, Leguminosae, Ericaceae, Asteraceae, Zingiberaceae	*Citrus* × *paradise*	[33]
	Hesperidin	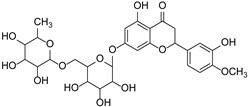		*Citrus aurantium*, *Citrus sinensis*	[34]
Dihydroflavonols	Silymarin (Silibinin, Aisosilibinin, Silidianin, Silychristin)	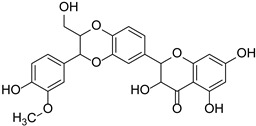	Asteraceae	*Silybum marianum*	[35]
Isoflavones	Daidzein	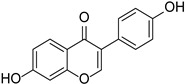	Papilionoideae, Iridaceae	*Pueraria lobata*	[36]
	Genistein	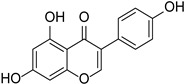		*Glycine max*, *Pueraria lobata*	[36,37]
	Soy isoflavone	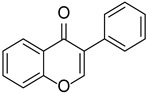		*Glycine max*	[38]
	Puerarin	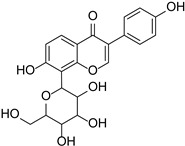		*Pueraria lobata*	[39]
	Biochanin A	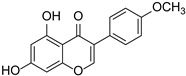		*Cicer arietinum*, *Trifolium pratense,* and *Glycine max*	[40]
Chalcones	Isoliquiritigenin	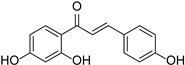	Asteraceae, Gesneriaceae, Leguminosae	*Glycyrrhiza*	[41]
	Licochalcone	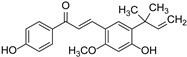		*Glycyrrhiza*	[42]
Flavan-3-alcohols	Epigallocatechin gallate	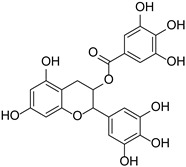	Theaceae	*Camellia sinensis*, *Theobroma cacao*	[43]
	Catechin	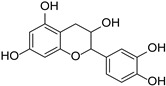		*Theobroma cacao*	[44]
Other flavonoids	Ginkgetin	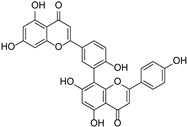	Ginkgoaceae	*Ginkgo biloba*	[45]
	Methylophiopogonanone A	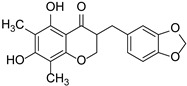	Liliaceae	*Radix Ophiopogonis*	[46]
	Swertiajaponin	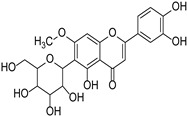	Gentianaceae	*Ziziphus jujuba*	[47]
	Scabiolide	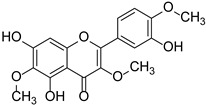	Asteraceae	*Centaurea cyanus*	[48]

### 3.2. Chemical Synthesis and Structural Modification

**Figure 3:**
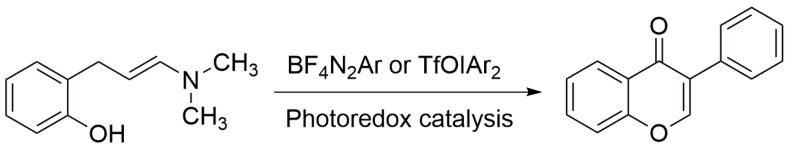
Photo-assisted synthesis of flavonoids.

As people become more conscious of health issues and the idea of “dual use of food and medicine” gains traction, flavonoids are increasingly utilized as food additives. The requirement for effective flavonoid synthesis is still growing since flavonoid extraction and separation from plants are insufficient to meet application needs. The main basic skeleton of flavanones, such as naringenin, pinocembrin, and eriodictyol, can be structurally modified to produce flavanones with different structures and functions. Flavonoids can be chemically synthesized using a variety of techniques, including the Auwers method, the Baker–Venkataraman (BKVK) method, the Algar–Flynn–Oyamada (AFO) method, the photocatalytic synthesis method, etc. However, most of the methods are limited by reason of cumbersome reactions and low yields. Nowadays, only the AFO and photocatalytic synthesis methods are widely used. Among them, photocatalytic synthesis has the advantages of green environmental protection and high synthesis efficiency. For this reason, researching photocatalytic flavonoid synthesis is crucial to the production and application of flavonoids. Moreover, the synthetic route for the synthesis of flavonols by the AFO method is the condensation of benzaldehyde derivatives with acetophenone to generate the raw material **2**′-hydroxychalcone, and the oxidative cyclization of **2**′-hydroxy chalcone with H_2_O in alkaline solution to generate flavonols. Mkrtchyan et al. generated flavonoids by replacing NH_4_SCN with BF_4_N_2_Ar and TfOIAr_2_, and irradiating them with blue and green light, respectively [49]. The specific process is shown in Figure 3.

**Figure 4:**
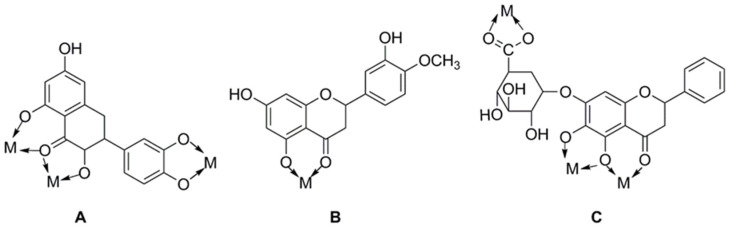
Metal chelates sites of common flavonoid compounds. (**A**) Quercetin; (**B**) diosmetin; (**C**) baicalin.

The main methods of chemical modification of flavonoids include the chelation of metal ions, the introduction of halogenated elements, and active groups. The physicochemical characteristics and biological activities of flavonoids can be directly influenced by changes in their chemical structure. Flavonoids have a strong chelating effect on metal ions, and the ligands of their complexes can synergize with metal ions to increase their biological activities. Many flavonoids such as rutin, quercetin, baicalin, etc., have been reported to be chelated with metal ions to affect their biological activities, among which the chelation of copper, zinc, and rare-earth metal elements with flavonoids has been reported more. Figure 4 shows some common flavonoid chelation sites with metal ions. It is possible to increase the biological activity of flavonoids by adding halogenated elements to them; these elements are primarily fluorine, chlorine, and bromine. The order of activity of introduced substances is usually fluorine greater than chlorine greater than bromine [50]. The introduction of reactive groups is mainly methyl, acyl, and hydroxyl groups. Different active groups can be added to flavonoid molecules to alter their shape, increase their biological activity, or even create entirely new physiological roles. Copmans et al. [51] investigated the effects of naringenin, kaempferol, and their three methylated derivatives on epileptic seizures (PTZ-induced), and the results showed that the methylated flavanone NRG-dm was very effective against PTZ-induced epileptic seizures in zebrafish larvae. The hydroxyl group can be added to flavonoids to increase their water solubility and antioxidant activity. The introduction site of the hydroxyl group has varying effects on flavonoid activity, and the relative activity and the introduction site are related in the following ways: 7 > 4′ ≈ 3 > 3′ >> 5 [52].

**Figure 5:**
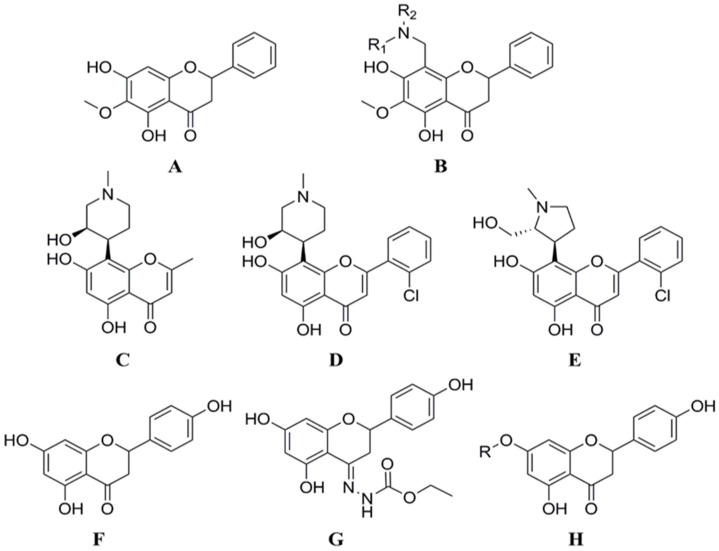
The synthesis or modification of flavonoids at the A and B rings. (**A**) Oroxylin A; (**B**) Compound 4 (8-((dimethylamino)methyl)-5,7-dihydroxy-6-methoxy-2-phenylchroman-4-one); (**C**) Rohitukine; (**D**) Flavopiridol; (**E**) P276-00 (5,7-dihydroxy-8-((2R,3S)-2-(hydroxymethyl)-1-methylpyrrolidin-3-yl)-2-o-tolylchroman-4-one); (**F**) Naringenin; (**G**) (E)-ethyl 2-(5,7-dihydroxy-2-(4-hydroxyphenyl)chroman-4-ylidene)hydrazinecarboxylate; (**H**) 5-hydroxy-2-(4-hydroxyphenyl)-7-methoxychroman-4-one.

From the perspective of chemical organic synthesis, the synthesis or modification of flavonoids at the C ring is the biggest challenge, and this is critical for the nature and characterization of the synthetic end products. On the other hand, the selective introduction of functional groups to the A and B rings has been a major scientific concern among chemical scientists in recent times. Li et al. structurally modified lignans by introducing amino methylene at the 8-position via a Mannich reaction to produce compound 4 (Figure 5B), which was evaluated for CDK1/cyclin B inhibitory activity. Among them, the IC_50_ value of **8**-N-methylpiperazinylmethylidene xylophilus against CDK1/cyclin B was 0.92 μmol/L [53]. Both of the two flavonoids derived from the natural product rohitukine (Figure 5C), flavopiridol (Figure 5D), and P276-00 (Figure 5E), had very strong CDK inhibitory effects [54,55], and are currently in the clinical research stage of oncology treatment for use. Yoon et al. designed and synthesized six derivatives modified at 7 or 4 positions of naringenin based on the naringenin structure (Figure 5F). In the inhibition assay of HCT116 cell proliferation and CDK2/cyclin E kinase activity, all six compounds revealed significantly higher activity than naringin (Figure 5G,H). Among them, the IC_50_ values of the compounds against HCT116 cells ranged from 1.20 to 20.01 μmol/L (IC_50_ of naringenin was 36.75 μmol/L). The inhibition rate of CDK2/cyclin E kinase by all naringenin derivatives at a concentration of 10 μmol/L ranged from 42.0% to 84.0% (naringenin’s inhibition rate was 14.9%). The enzyme inhibitory activity was consistent with the anti-proliferation activity of tumor cells. The molecular docking results indicated that naringenin derivatives bind adequately to CDK2/cyclin E compared with naringenin. Furthermore, the introduction of a bulky group at the 7-position of naringenin or a nitrogen-containing functional group at the 4-position can enhance its anti-proliferation activity in HCT116 cell [56].

By adding aryl functional groups and gallic acid derivatives, Murad et al. created and synthesized a variety of flavonoid derivatives. Then, nuclear magnetic resonance and infrared spectroscopy were used to confirm the target compounds’ structures. Pharmacodynamic assays showed that compound 3 effectively inhibited the proliferation of Caco-2 cells (IC_50_ = 2.42 μg/mL), and compound 4 possessed antioxidant activity (IC_50_ = 3.53 ± 0.1 μg/mL) and selectively inhibited COX-2 (IC_50_ = 6.02 ± 0.33 μg/mL). It can be seen that the chemical modification of flavonoids can improve their biological activities [57]. For example, 8-chloro-3′,4′,5,7-tetrahydroxyflavone has been reported to modulate the oxidative burst of phorbol myristate acetate-activated neutrophils more efficiently than its parent compounds, and is therefore an alternative anti-inflammatory therapy [58].

### 3.3. Biosynthesis

Flavonoids are one of the major metabolites of plant endophytic fungi [59,60]. In recent years, the isolation of flavonoid-producing endophytic fungi from plants and analysis of their biosynthetic pathways have been one of the research hotspots. The metabolites of the endophytic fungi of *Portulaca oleracea* were initially identified, based on the chromogenic reaction of flavonoids and the characteristics of the ultraviolet spectrum. Through the 18S rDNA sequence amplification of the isolated strain AG-10, combined with the morphological characteristics of the colony and conospores, the endophytic fungal were identified as *Fusarium* sp. [61]. A total of 116 strains of endophytic fungi were isolated from *Ginkgo biloba* roots and stem via the tissue culture method. The metabolites of endophytic fungi were investigated using chromogenic reaction and HPLC. Finally, two endophytic fungi that can produce flavonoids were selected. The total flavone yield in the fermentation broth was determined by spectrophotometry, which was more than 20 mg. The phylogenetic analysis of morphological characteristics and internal transcribed spacer (ITS) of the fungi indicated that the two strains belong to the genus of *Penicillium* and *Mucor*, respectively. It is the first time that *Penicillium* has been reported to be a flavonoid-producing endophytic fungus of *Ginkgo biloba* [62]. Zhou et al. used the roots, stems, and leaves of *Cinnamomum camphora* in spring as materials to isolate, purify, and screen functional strains capable of producing flavonoids by tissue culture method. A total of 10 strains of endophytic fungi were isolated with flavonoid-producing function, among which strain YZ-29 had relatively strong flavonoid-producing ability [63]. Cheng et al. isolated an endophytic fungus Gs-6 from *Gentiana straminea* and found that the yields of isovitexin, quercetin, and isoorientin were 0.824 mg/L, 1.110 mg/L, and 1.569 mg/L, respectively. The endophytic fungus Gs-6 was identified as *Cadophora* sp. via morphological and molecular identification [64]. In addition, a flavonoid-producing endophytic fungus YS101 from *Saussurea involucrata* was identified by chromogenic reaction and thin-layer chromatography (TLC) screening and was identified as *Aspergillus tabacinus*. The content of rutin in 200 mL potato dextrose agar (PDA) medium was determined by HPLC to be more than 9.0 μg (Table 3) [65].

**Figure 6:**
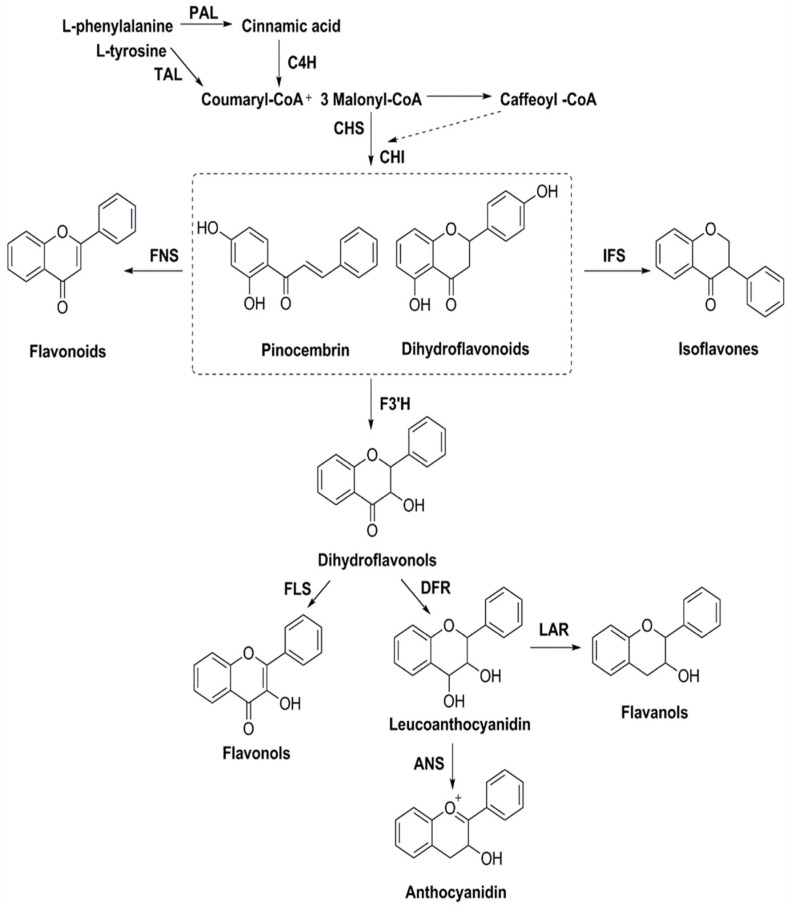
Biosynthesis pathways of flavonoids.

**Figure 7:**
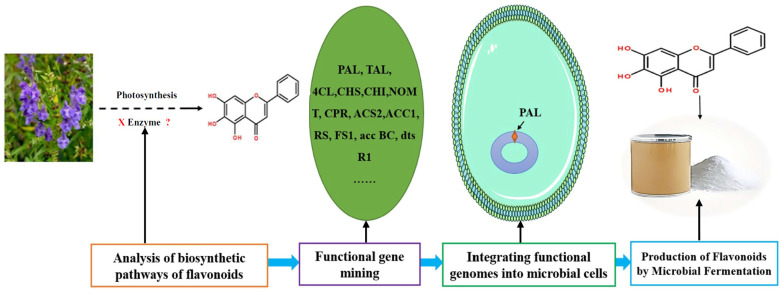
Microorganisms synthesis strategy of flavonoids using genetic engineering technology.

The biosynthesis of flavonoids has been studied deeply, and its biosynthesis pathways involve the acetate pathway (A-ring synthesis) and the shikimate pathway (B-ring and intermediate C3 synthesis). Specifically, the A-ring is synthesized via the acetate pathway from three malonyl-CoA molecules generated by the conversion of glucose, and the B-ring is synthesized via the shikimate pathway from 4-coumaroyl-CoA generated from phenylalanine [66]. The A and B rings are condensed to chalcone by chalcone synthase (CHS), and chalcone is then catalyzed by chalcone isomerase (CHI) to produce dihydroflavone. Dihydroflavonoids are the common precursors of most flavonoids, which can be catalyzed by flavonol synthase (FNS), isoflavone synthase (IFS), flavonoid 3′-hydroxylase (F3′H), and other enzymes to generate other flavonoids. Enzymes included in the biosynthesis of flavonoids include phenylalanine ammonia-lyase (PAL), tyrosine ammonia-lyase (TAL), cinnamic acid-4-hydroxylase (C4H), coumaroyl-CoA (4CL), CHS, CHI, FNS, IFS, F3′H, and so on (Figure 6). *Ampelopsis grossedentata* is a kind of medicinal and edible plant resource with strong health effects that is rich in flavonoids, amino acids, vitamins, and many kinds of microelements [67,68]. Liu et al. used RT-PCR technology to clone the main coding gene *CYP73A* of trans-cinnamate 4-monooxygenase (C4H), which is a key enzyme in the synthesis pathway of flavonoids. Furthermore, they found that this gene plays an important role in the metabolic pathway in flavonoids of *Ampelopsis grossedentata*, which lays a theoretical foundation for the construction of the overexpression vector of the *CYP73A* gene and its genetic transformation system, and the realization of the further high-efficiency expression of flavonoids in the medicinal materials of *Ampelopsis grossedentata* [69]. Analyzing the biosynthetic pathway of endophytic flavonoid-producing fungi, mining key functional genes as well as assembling the synthetic pathway into microbial cells to realize microbial fermentation to produce flavonoids is one of the most efficient methods of flavonoid production at present (Figure 7). Jin et al. discovered, identified, and analyzed two key oxygen-methyltransferases, CsFAOMT1 and CsFAOMT2, that catalyzed the biosynthesis of methylated catechin by constructing genetically isolated populations and using multiomics techniques, revealing the biosynthesis mechanism of methylated catechin derivatives in tea trees [70]. Wang et al. constructed an *E. coli* that can synthesize baicalein or nobaicalein in a targeted manner by providing only two different precursors, phenylalanine or tyrosine. The team first transferred six genes of enzymes such as 4CL and FNS I from parsley, PAL from red yeast, CHS from petunia, and CHI from *Medicago sativa* into yeast constructed via the metabolic pathway of apigenin, an important intermediate of flavonoids. Then, the heterologous synthesis of baicalin and nobaicalein was achieved by cloning *F6H* from *Scutellaria baicalensis* and *AtCPR* from *Arabidopsis thaliana*. On this basis, the team made *E. coli* ultimately produce 23.6 mg/L of baicalein and 106.5 mg/L of nobaicalein by overexpressing the malonyl coenzyme A synthesis gene *acs* as well as the fatty acid synthesis gene *fabF*, and by introducing the malonyl coenzyme A synthase gene *matB* and the malonate carrier protein gene *matC* of the *Rhizobium trilobatum* [71]. Cathie Martin’s team, a British expert in plant metabolic engineering, overexpressed *Arabidopsis* transcription factors such as AMYB12 in *Solanum lycopersicum* and dramatically increased the content of flavonoids and other substances in *Solanum lycopersicum*, with a dry matter mass fraction of 100 mg/g [72]. After introducing the transcription factors Delila and Rosea1 derived from *Antirrhinum majus* into *Solanum lycopersicum*, Tohge et al. [73] detected an increase in the content of anthocyanins and phenylpropyl flavonoid derivatives. The team then introduced Arabidopsis-derived flavonoid regulator AtMYB12 into *Solanum Lycopersicum* and detected that the mass fraction of flavonoids and ethyl phydroxycinnamate reached 10% of the dry mass of the fruit. Rodriguez et al. reported the development of a novel yeast cell factory for de novo production of flavonoids from glucose, and obtained 26.57 ± 2.66 mg/L of kaempferol in yeast [74]. A modular co-culture method was performed to divide the synthesis pathway of sakuranetin into two modules and construct the related genes in *E. coli*., where glucose was used as the substrate to expand cultivation in an intermittent reactor, which led to the production of 79.0 mg/L of sakuranetin [75]. An efficient biocatalytic cascade consisting of *Trollius chinensis* C-glycosyltransferase (TcCGT) and Glycine max sucrose synthase (GmSUS) was performed for the production of orientin and vitexin using a fed-batch operation strategy. The maximal titers of orientin and vitexin reached 7090 mg/L and 5050 mg/L, respectively, with corresponding molar conversions of 98.7% and 97.3%, respectively (Table 4) [76].

Even though there has been significant progress in producing flavonoids by biosynthesis, the efficiency of flavonoid biosynthesis is still severely limited by the intricacy of natural product metabolic pathways and the limitations of current analytical methods. Furthermore, issues like the low expression effectiveness of genetic components in plants following their transfer into heterologous microorganisms impede the advancement of flavonoid biosynthesis techniques.

**Table 3:** Flavonoid-producing endophytic fungi and their host plants.

Endophytic Fungi	Host Plant	References
*Fusarium* sp.	*Portulaca oleracea*	[61]
*Penicillium* and *Mucor*	*Ginkgo biloba*	[62,77]
*Leptosphaeria*, *Fusarium* sp.	*Gentiana straminea*	[64]
*Alternaria*	*Erigeron breviscapus*	[78]
*Phomopsis*	*Cupressus funebris*	[79]
*Aspergillus*	*Opuntia stricta*, *Saccharum officinarum*	[80,81]
*Chaetomium* sp., *Colletotrichum* sp.	*Conyza blinii*	[82]
*Pestalotiopsis*	*Rhizophora mucronata*	[83]
*Phomopsis longicolla*	*Dicerandra*	[84]
*Penicillium*	*Carica papaya*	[85]
*Mycelia sterlia*	*Vaccinium vitisidaea*	[86]
*Chaetomium*	*Opuntia*	[87]
*Preussia funiculata*	*Stellera chamaejasme*	[88]
*Aspergillus tabacinus*	*Saussurea involucrata*	[69]
*Alternaria tenuissima*, *Dothiorella gregaria*, *Penicillium aethiopicum*, *Nothophoma quercina,* and *Hypoxylon perforatum*	*Loranthus tanakae*	[59]
*Alternaria*, Eupenicillium	*Cyclocarya paliurus*	[89]
*Fusarium* sp., *Penicillin pinophilum*	*Apocynum venetum*	[90]
*Sordariomycetes* sp., *Hypoxylon fragiforme*, *Phanerochaete magnoliae*, *Daldinia eschscholtzii*	*Dendrobium officinale*	[91]
Not identified	*Glycyrrhiza uralensis*	[92]

**Table 4:** Genetic engineering synthesis and yield of flavonoids from microorganisms.

Precursor	Expression Vector	Key Enzyme/Gene	Product	Yield (mg/L)	References
Glucose	*Y. lipolytica*	Chalcone synthase (CHS), Cytochrome P450 reductases (CPR)	Naringenin	252.4	[93]
Tyrosine and malonate	*E. coli*	Chalcone synthase (CHS)		191.9	[94]
Glucose	*S. cerevisiae*	*4CL3*, *CHS1*, *CHI1*, *C4H*, *CPR*, *CHS3*, *PAL1*, *TAL1*		112.9	[95]
Glucose	*Yarrowia*	*TAL*, *4CL*, *CHS*, *CHI*, *F3′H*, *CPR*, *ACS2*, *ACC1*		71.2	[96]
Coumaric acid	*S. venezuela*	*CCL*, *CHS*		4	[97]
Phenylalanine	*E. coli*	*acc BC*, *dts R1*	Pinocembrin	58	[98]
Daidzein	*E. coli*	S-adenosine-l-methionine synthase (SAMS)	4′-O-methyl daidzein	102.88	[99]
Glucose	*Y. lipolytica*	Chalcone synthase (CHS), Cytochrome P450 reductases (CPR)	Eriodictyol	134.2	[93]
Kaempferol	*E. coli*	Glycosyltransferase C	Afzelin	1900	[100]
Naringenin	*E. coli*	Glycosyltransferase	Astragalin	1738.5	[101]
L-tyrosine	*E. coli*	*PAL*, *sc CCL*, *CHS*, *CHI*, *FS1*, *acc BC*, *dts R1*	Apigenin	13	[102]
L-tyrosine	*E. coli*	*PAL*, *Sc CCL*, *CHS*, *CHI*	Kaempferol	15.1	[102]
Naringenin	*E. coli*	*Cisf3H*, *C3H*, *Cufls*, *CLS*		1184.2	[101]
Catechin and Glucose	*E. coli*	Anthocyanidin synthase (PhANS), Cyanidin 3-O-glucosyltransferase (At3GT)	Cyanidin 3-O-glucoside	439	[103]
Phenylalanine	*S. cerevisiae*	*PAL*, *CPR*, *C4H*, *4CL*, *CHS*, *CHI*, *F3′H*, *FLS*	Quercitrin	-	[104]
Quercetin	*E. coli*	Glycosyltransferase C (GtfC), the dTDP-rhamnose synthesis genes (rmlABCD), glucan 1,4-alpha-maltohexaosidase		4300	[95]
Hesperetin	*E. coli*	Anthocyanidin synthase (PhANS), cyanidin 3-O-glucosyltransferase (At3GT)	Hesperetin-3′-O-rhamnoside	2400	[95]
Glucose, L-tyrosine	*E. coli*	*TAL*, *4CL*, *CHS*, *CHI*, *acc BC*, *dts R1*, *DE3*	Eriodictyon	107	[105]
Cumaric acid	*E. coli*	*4CL1*, *STS*, *ACC*, *Bir A*	Resveratrol	910.9	[106]
Glucose	*E. coli*	*TAL*, *4CL*, *CHS*, *NOMT*, *pps A*, *tkt A*, *aro Gfbr*, *yr Afbr*	Sakuranetin	40.1	[107]
Caffeic acid	*E. coli*	*4CL1*, *STS*	Piceatannol	13.3	[108]
Genistein	*E. coli*	S-adenosine-l-methionine synthase (SAMS)	4′-O-methyl genistein	46.81	[94]
Apigenin	*E. coli*	glycosyltransferase, sucrose synthase	Isovitexin	3772	[109]
Tyrosine	*E. coli*	Flavone C-6 hydroxylase(F6H), Partner P450 reductase from *Arabidopsis thaliana* (AtCPR)	Scutellarein	106.2	[71]
Luteolin	*E. coli*	Glycosyltransferase, Sucrose synthase	Isoorientin	3820	[109]
Glucose	*Yarrowia*	*TAL*, *4CL*, *CHS*, *CHI*, *F3′H*, *CPR*, *ACS2*, *ACC1*	Taxifolin	48.1	[96]
	*Y. lipolytica*			110.5	[93]
	*S. cerevisiae*			336.8	[110]
Phenylalanine	*E. coli*	*PAL*, *Sc CCL*, *CHS*, *CHI*, *CHI*, *FS1*, *acc BC*, *dts R1*	5,7-dihydroxyflavone	58	[98]
Xylose	*S. cerevisiae*	*ARO4*, *ARO7*	p-Coumaric acid	242	[111]
Apigenin and luteolin	*E.coli-E.coli coculture*	Trollius chinensis C-glycosyltransferase (TcCGT), Glycine max sucrose synthase (GmSUS)	Orientin	7090	[76]
			Vitexin	5050	[76]

## 4. Evaluation of Biological Activity and Druggability

### 4.1. Antioxidant Effects

Flavonoids exhibit strong antioxidant and free radical scavenging properties. Among them, the antioxidant activity of quercetin through its effects on glutathione enzyme activity, oxidative stress signaling pathways, and reactive oxygen species (ROS) has been widely recorded [112]. Naringin and apigenin can enhance the antioxidant capacity of the skin by reducing the levels of malondialdehyde and lipid peroxide, and increasing the catalase and total antioxidant capacity [113]. Total flavonoids from *Acer truncatum* leaves have been shown to be able to prevent oxidative stress caused by juglone by upregulating catalase superoxide dismutase activity and lowering malondialdehyde and ROS concentrations [114]. The in vitro antioxidant activity of flavonoid-rich ethanolic extract (EE) was examined and it was found that EE possessed good scavenging ability against **2**-diphcnyl-l-picrylhydrazyl (DPPH), **2**,**2**′-azinobis (**3**-ethylbenzothiazoline-**6**-sulfonic acid ammonium salt (ABTS), and hydroxyl radicals with IC_50_ values of 296.95 ± 13.24 μg/mL, 94.31 ± 9.13 μg/mL, and 9.21 ± 0.15 mg/mL, respectively. The EE was able to drastically lower intracellular ROS levels and block glucosidase activity in the high glucose-induced L02 cell model, which attenuated the inflammatory response and oxidative stress-induced cellular damage [115]. In addition, the results of the antioxidant activity assay showed that the total flavonoid glycosides (FG) extracted from *Camellia oleifera* had good DPPH radical scavenging ability and protected against H_2_O_2_-induced oxidative damage in vascular endothelial cells [116]. In rat liver microsomes, **6**″-O-Acetylgenistin and **6**″-O-Acetyldaidzin significantly suppressed lipid peroxidation with IC_50_ values of 10.6 and 8.2 μM, respectively [117].

### 4.2. Anti-Inflammatory and Analgesic Effects

Many studies have demonstrated the significant anti-inflammatory and analgesic biological activities of flavonoids, such as rutin, hydroxyrutin, and dihydroquercetin, among others. These flavonoids have been shown to have clear inhibitory effects on carrageenan, 5-hydroxytryptamine (5-HT), prostaglandin E2 (PGE2)-induced edema of the rat paw, formaldehyde-induced arthritis granuloma, and other related conditions. Luteolin, hypericin, rutin, quercetin, and *Ginkgo biloba* total flavonoids have good analgesic effects. Luteolin has potent anti-inflammatory activity in vitro as well as in vivo, and its derivative luteolin-**3**′-O-phosphate has similar anti-inflammatory activity and has demonstrated superior anti-inflammatory effects in a variety of experiments, as well as inhibiting mitogen-activated protein kinase (MAPK) and nuclear factor-kappa B (NF-κB) more effectively than luteolin [118]. Negletein showed significant anti-inflammatory activity; it can inhibit the secretion of TNF-α and IL-1β with IC_50_ values of 16.4 μM and 10.8 μM, respectively. Sappanone A is a homoisoflavanone that exerts a mitigating effect on allergic airway inflammation in ovalbumin-induced asthma. In addition, sappanone A significantly inhibited LPS (HY-D1056)-induced nitric oxide production and exerted its anti-inflammatory effects by modulating NF-κB and heme oxygenase 1(HO-1)/nuclear factor erythroid 2-related factor 2 (Nrf2) signaling pathways in BV2 and RAW264.7 cells [119]. Pectolinarin exerts its anti-inflammatory effects by inhibiting the secretion of IL-6 and IL-8 and the production of PGE2 and nitric oxide (NO). Hesperidin methylchalcone inhibits the production of cytokines and the activation of NF-κB, and thus possesses anti-inflammatory, antioxidant, and analgesic effects. Kaempferol **3**-O-β-D-glucuronide inhibits various proinflammatory mediators including IL-1β, NO, PGE2, and leukotrienes B4 (LTB4), and promotes the secretion of anti-inflammatory cytokine IL-10. Broussochalcone A inhibits iron-induced lipid peroxidation and NO synthesis in LPS-activated macrophages [120]. In vivo and in vitro experiments revealed that quercetin deactivates the NF-κB pathway by inhibiting the phosphorylation of IκB protein, and further inhibits the ability of LPS and interferon-gamma (IFN-γ) to process DNA in microglia [121,122]. It has also been reported that quercetin and apigenin and their metabolites were able to reduce the expression of miR-155 and downregulate the activities of proinflammatory factors such as tumor necrosis factor-α (TNF-α), IL-6, and IL-1β, thus exerting an anti-inflammatory effect [123]. In BV-2 microglia, the LPS-induced phosphorylation of NF-κB/IκB, Toll-like receptors 4 (TLR4)/myeloid differentiation primary response 88 (MyD88), and the phosphor-p38 mitogen-activated protein kinases (p38MAPK)/c-Jun N-terminal kinase (JNK) signaling pathway was inhibited after the treatment of **6**-methoxyflavone (**6**-MeOF). Meanwhile, **6**-MeOF also inhibited the LPS-induced increase in the contents of NO, ROS, iNOS, and COX-2, and promoted the expression of HO-1 and NAD(P)H: quinone oxidoreductase 1(NQO1). In vivo experiments also confirmed that 6-MeOF can inhibit *LPS*-induced NO production and has anti-inflammatory and antioxidant potential [124]. Hydrogenated isoflavones reduced the production of LPS-stimulated proinflammatory mediators and enzymes, including TNF-α, IL-6, NO, iNOS, and COX-2 [125]. Eupatilin could increase SOD, GSH, and IL-10 levels, and decrease the contents of MDA, TNF-α, IL-1β, and IL-6, as well as significantly downregulating the expressions of the NF-κB signal pathway, thereby relieving the inflammatory responses [126]. Using transcriptomic techniques, the anti-inflammatory mechanisms of flavonoids in Abrus mollis and Abrus cantoniensis were examined. The findings indicated that the flavonoids either activated or downregulated genes related to inflammation [127].

### 4.3. Anti-Tumor Effect

One area of scientific interest for the pharmacological activity of flavonoids is the investigation of anticancer activity. Linarigenin could arrest the cell cycle of human lung cancer A549 cells and human liver cancer HepG2 cells by increasing the expression of CDK endogenous inhibitors, *p53* and *p21* genes [128]. Gupta et al. found that apigenin could cause human prostate cancer LNCaP cells to arrest in the G1 phase and significantly reduce the expression of cyclin D1, D2, E, and CDK2, 4, and 6, as well as induce the expression of p21 and p27 [129], and apigenin induced the expression of p-p53, p53, p21, and p27 in human bladder cancer T-24 cells, which made cyclin A, B1, E, CDK1, 2, and Cdc25C levels decrease, blocking the cells in the G1 phase, thus blocking the cell cycle process and inhibiting tumor cell proliferation [130]. Hsu et al. showed that baicalein could downregulate the expression of cyclin D2, A, CDK1 and 2, and upregulate the expression of p15, p21, p53, and cyclin E in murine cardiac endothelial cells, which caused endothelial cells to stagnate in the G1 and G2 phases [131], and baicalein was able to induce the apoptosis of lung cancer CH27 cells and caused lung squamous cancer cells to stagnate in the S-phase through the downregulation of the CDK4, the cyclin B1, and D1 expression [132]. Lien et al. indicated that Nobiletin inhibited the growth of human U87 and Hs683 glioma cells, causing them to be arrested in the G0/G1 phase. The method of action could involve downregulating the production of cyclin D1, CDK2, 4, and E2F1 in order to inhibit the protein kinase B (Akt) and MAPK signaling pathways. In addition, nobiletin has been found to inhibit tumor cell growth by suppressing glioma cell migration [133].

Soybean isoflavones form a class of flavonoids in *Glycine max* and its products, mainly including daidzin (**7**,**4**′-dihydroxyisoflavone) and genistin (**5**,**7**,**4**′-trihydroxyisoflavone). They can produce estrogen-like activity that is protective against breast cancer only when they are decomposed by bacteria or hydrolyzed into daidzein and genistein in the stomach [134]. The latest research has shown that the anticancer effect of soybean isoflavones is not entirely estrogen-like, such as isoflavones interfering with the expression of the two copper transporter genes, CTR1 and ATP7A, in cancerous cells [135]. In addition to acting as antioxidants to stop DNA oxidative damage, soybean isoflavones can also stop tumor growth by causing tumor cells to die and preventing the production of oncogenes. In conclusion, soybean isoflavones have a variety of mechanisms of action that may make them effective as adjuvant chemotherapy and have a good impact on the treatment of cancers like ovarian cancer [136,137].

### 4.4. Anti-Anxiety Effect

Flavonoids can be utilized to treat anxiety disorders because of their effect on the central nervous system [138]. It was shown that hydroxylic extract of *Spinacia oleracea* (mainly flavonoids) given to EPM anxiety model mice at a dose of 200 mg/kg was comparable to the anxiolytic drug diazepam (2 mg/kg) [139]. Today, 15 natural flavonoid monomer compounds with anxiolytic effects have been recorded, including: chrysin, apigenin, apigenin-**7**-glucose, wogonin, baicalein, baicalin, luteolin, **6**-hydroxyflavones, amentoflavone, spinosin, etc. The common structural feature of most of the flavonoids with anxiolytic effects is the almost simultaneous hydroxyl group at the 5, 7 position of the A ring (except for 6-hydroxyflavones), such as chrysin. The difference lies in whether there are hydroxyl or methoxy substitutions at the 6, 8 positions of the A ring and the 3′, 4′ positions of the B ring. A total of 1 mg/kg of chrysin significantly increased the number and duration of rats entering the open arm in the elevated cross-maze experiment, while 3 mg/kg of chrysin increased the number and duration of rats exploring the hole in the whole plate experiment [140]. It is evident that chrysin has an anti-anxiety effect and, at an effective dosage, has no effect on muscle relaxation. Kaempferol administered orally at doses higher than 0.02 mg/kg showed significant anti-anxiety activity through the elevated cross-maze experiment in mice [141]. Aguirre-Hernández et al. revealed that quercetin possesses anti-anxiety activity [142]. In addition, Vissiennon et al. demonstrated the anti-anxiety activity of kaempferol (1.0 mg/kg) and quercetin (0.5, 1 mg/kg) through an elevated cross-maze experiment, and the anti-anxiety effect of myricetin was not significant [143]. Acute and chronic administration of ellagic acid to mice has produced an anti-anxiety-like effect in the elevated plus-maze test [144].

### 4.5. Cardiovascular Protective Effect

When it comes to controlling endothelium function and preventing and treating cardiovascular illnesses, flavonoids exhibit strong pharmacological action and promise as dietary supplements and pharmaceutical ingredients [145]. Higher dietary intakes of flavonoids may be beneficial to the prevention of cardiovascular diseases [146]. During the process of clearing ROS, rutin can be oxidized to quinone derivatives, which can upregulate Nrf2 mediated endogenous antioxidant response and selectively exert its protective effect in areas of increased oxidative stress, thereby reducing the risk of cardiovascular disease(CVD) [147]. Hesperidin, as an antioxidant and anti-inflammatory agent, reduces serum levels of triglyceride in depressed postcoronary artery by-pass grafting (CABG) patients, is also effective in preventing the progression of atherosclerosis in CVD patients after CABG surgery [148]. Chrysin can lessen ventricular failure, hemodynamic dysfunction, and myocardial ultrastructural damage brought on by isoproterenol in rat models. It can also lessen collagen white expression in myocardial infarction and fibrosis in the interstitial and perivascular areas [149]. Genistein significantly reduced the oxidized low-density lipoprotein (ox-LDL)-induced monocyte chemoattractant protein-1 (MCP-1), vascular cellular adhesion molecule-1 (VCAM-1), and intracellular adhesion molecule-1 (ICAM-1) secretion and mRNA transcription on human umbilical vein endothelial cells, and reversed atherosclerotic injury caused by angiotensin-induced phosphorylation [150].

### 4.6. Antidiabetic Effect

Diabetes is considered to be one of the three major diseases that seriously endanger human health [151]. It is an illness with a complicated etiology that affects several body organs. Flavonoids’ wide range of pharmacological effects have made them a popular research subject for natural hypoglycemic medications [152]. Fisetin was discovered to be able to suppress the expression of the genes phosphoenolpyruvate carboxykinase (PEPCK) and glucose-6-phosphatase (G-6-Pase), which are the key enzymes of hepatic gluconeogenesis, in order to maintain glucose homeostasis and improve insulin resistance. Fisetin was also found to be able to lower blood glucose, increase the amount of hepatic glycogen, and enhance the activities of hexokinase, pyruvate kinase, glucose-6-phosphate dehydrogenase, and fructose 1,6-bisphosphatase during the process of glycolysis in diabetic rats [153,154]. Quercetin and kaempferol exert hypoglycemic effects mainly by inhibiting intestinal starch digestion and hepatic glucose production, promoting glucose uptake by skeletal muscle, and protecting pancreatic cells from damage. Zang et al. reported that apigenin enhances the phosphorylation of AMPK in HepG2 hepatocytes, and the effect is 200 times more effective than that of metformin [155]. Moreover, it can suppress the phosphorylation of acetyl coenzyme A carboxylesterase (ACC) and reduce the accumulation of cellular lipids in a high-glucose environment. In addition, mitochondrial dysfunction is an important causative factor in the development of insulin resistance. Apigenin could protect deoxy-D-ribose from toxic effects and mitochondria from damage in HIT-T15β cells [156]. Similarly, apigenin and luteolin inhibit NF-κB activation and iNOS expression, and prevent IL-1β and IFN-γ-mediated inhibition of insulin secretion [157]. *Belamcanda chinensis* leaf extract has been shown to have a good anti-diabetic effect. The extract was used to identify 13 flavonoids, including swertisin and mangiferin [158]. Among these are the ability of swertisin to increase insulin secretion, convert excess glucose to fructose, and block the activity of aldose reductase in the polyol pathway [159].

The antidiabetic effects of apigenin, lignans, and baicalein in high-glucose and dexamethasone-induced insulin-resistant (IR) HepG2 cells. The results showed that all three flavonoids increased glucose consumption and glycogen synthesis in IR-HepG2 cells by activating glucose transporter protein 4 (GLUT4) and phosphor glycogen synthase kinase (GSK-3β). These flavonoids significantly inhibited the production of ROS and advanced glycosylation end products (AGEs), which were closely related to the inhibition of phosphorylated forms of NF-κB and P65. Furthermore, in IR-HepG2 cells, the expression levels of the insulin receptor substrate-1 (IRS-1), insulin receptor substrate-2 (IRS-2), and phosphatidylinositol 3-kinase (PI3K)/Akt pathways were partially activated by flavonoids, and these pathways were variably active [160].

### 4.7. Antibacterial Activity

It is crucial to find a new batch of antibacterial compounds from natural pharmaceuticals due to the severity of bacterial antibiotic resistance and the rise of “super bacteria”. Wang et al. used semi-preparative HPLC for the isolation and purification of flavonoids from *Ziziphus jujuba*, and a total of five flavonoids including epicatechin, quercetin, rutin, isoquercetin, and hyperin were identified using nuclear magnetic resonance spectroscopy (NMR). Further antimicrobial activity assays showed that quercetin had the greatest inhibitory effect on *E. coli*, *Shigella*, and *P. aeruginosa*, with the minimum inhibitory concentration (MIC) of 250 μg/mL against *E. coli* or *P. aeruginosa*, and the MIC of 125 μg/mL against *Shigella*. The inhibitory effect of rutin on *E. coli* was the highest, with an MIC of 62.5 μg/mL. The MIC of hyperin was 250 μg/mL for Bacillus subtilis and 62.5 μg/mL for *E. coli*. Furthermore, research has demonstrated that flavonoids have a substantial antibacterial impact at pH values below 6 and that this effect dramatically diminishes as pH rises. Five metal ions including Na^+^, Ca^2+^, K^+^, Fe^2+^, and Mg^2+^ generally increased the antibacterial activity of quercetin against *E. coli*, *Shigella*, and *P. aeruginosa*, and rutin against *E. coli*. Moreover, Ca^2+^ and K^+^ had the most significant enhancement effect on hypericin antibacterial activity. According to studies, flavonoids derived from Ziziphus jujuba are thought to be a potential natural antibacterial that have use in the food and pharmaceutical sectors [161]. Jacquelyn et al. indicated that gancaonin G has antibacterial activity against *Streptococcus* mutants and MRSA strains [162]. As a result, flavonoids that target drug-resistant bacteria will continue to be identified in greater numbers in the future and will be a continued focus of research for a while [163].

### 4.8. Therapeutic Effect on Alcoholic Fatty Liver and Non-Alcoholic Fatty Liver

Since the prevalence of non-alcoholic fatty liver disease (NALFD) and alcoholic fatty liver disease (AFLD) has been rising recently, dihydromyricetin is a useful treatment for ethanol intoxication symptoms and for minimizing the harm that ethanol does to the liver. In the US, dihydromyricetin is currently utilized as a dietary supplement to avoid hangovers from alcohol [164]. An intraperitoneal injection of dihydromyricetin (5, 10 mg/kg) in alcohol-fed alcohol-related liver disease (ALD) mice can significantly increase the expression of enzymes related to ethanol metabolism and lower the levels of serum inflammatory cytokines and chemokines [165]. Baicalein could effectively attenuate ethanol-induced liver injury and improve the antioxidant capacity of ALD rats [166]. In mice with alcohol-induced liver injury, vitexin can significantly improve the abnormal elevation of serum AST, ALT, TC, TG, and TBIL as well as the pathological morphological changes in the liver. It can also normalize the uric acid, blood lipid, and aminotransferase indexes in ALD mice [167]. Currently, no drug has been approved for the treatment of NAFLD. Cao et al. studied the mitigating effect of quercetin on NAFLD and its mechanism of action by employing chemical inhibitors of autophagosome (**3**-methyladenine, **3**-MA), autolysosome (chloroquine, CQ), AMPK (Compound C, CC), and SIRT1 (selisistat, EX-527) using in vitro and in vivo models. The findings indicated that AMPK-mediated mitophagy is the mechanism via which quercetin reduces NAFLD [168].

### 4.9. Therapeutic Effect on Pulmonary Fibrosis

Multiple causes contribute to the widespread, persistent, and progressive interstitial lung disease known as pulmonary fibrosis. Normal lung tissue eventually undergoes structural alterations due to pulmonary fibrosis, which has a high mortality rate and considerable heterogeneity. At the moment, pirfenidone and nitidine are licensed to treat pulmonary fibrosis; nevertheless, more potent medications have to be created [169]. Studies have shown that the adenosine A2a receptor (A2aR) is a novel inflammatory regulator and baicalin can exert potent antifibrotic effects by activating A2aR. From the perspective of mechanism, the activation of A2aR inhibited BLM-induced lung fibrosis by suppressing tubuloglomerular feedback β1(TGF-β1) activation and downregulating the expression of extracellular-regulated protein kinases (ERK1/2) [170]. Baicalein may also cause cells to move from the G0/G1 phase to the S and G2/M phases, prevent the expression of the cell cycle proteins A, D, and E, stop fibroblast growth, and raise intracellular Ca^2+^ concentration [171]. Other studies have shown that the mechanism of baicalin’s inhibitory effect on pulmonary fibrosis may inhibit the TGF-β1/Smad signaling pathway by promoting the expression of recombinant Sirtuin 3 (Sirt3) in vivo [172].

Morin, as a kind of flavonoid compound isolated from the *Moraceae* plant, can significantly inhibit the transformation of fibroblasts into myofibroblasts and improve the pathological changes induced by bleomycin in mice. It was also shown that morin has inhibitory effects on TGF-β1-stimulated NIH-3T3 cells [173]. Silymarin decreases lung hydroxyproline concentrations and collagen fiber deposition, which have a substantial impact on pulmonary fibrosis. In addition, silymarin significantly altered the levels of key molecular markers, including decreased levels of TGF-β1 and fibronectin and increased levels of matrix metallopeptidase 2 (MMP-2) and IFN-γ [174]. Calycosin enhanced the expression of the Nrf2/HO-1 signaling pathway and induced apoptosis, and significantly inhibited bleomycin(BLM)-induced inflammation and collagen deposition in mice. The possible mechanism of action is that calycosin upregulates the expression of microtubule-associated protein1 light chain 3 (LC3), beclin1, and PTEN-induced putative kinase 1 (PINK1), decreases the expression of p62, and enhances autophagy by promoting the expression of lysosome-associated membrane proteins1 (LAMP1) as well as transcription factor EB (TFEB), which inhibits the release of lysosomal enzymes from ruptured lysosomes [175]. According to a different study, calycosin’s ability to mitigate pulmonary fibrosis was achieved via controlling the miR-375/Yes-associated protein 1(YAP1) signaling pathway, which prevented TGF-β1-induced epithelial–mesenchymal transition (EMT) [176]. Ginkgetin extracted from *Ginkgo biloba* exerts ameliorative effects on oxidative stress and lung fibrosis, primarily through an AMPK-dependent signaling pathway [45]. 

Furthermore, it is becoming clear that mitochondrial dysfunction plays a significant pathogenic role in the development of pulmonary fibrosis, while the exact processes behind this failure are as yet unknown. Consequently, the research into how flavonoids regulate mitochondrial activity in pulmonary fibrosis will be crucial.

### 4.10. Other Biological Effects

In the development of natural products, flavonoids represent a class of potentially important resources for drug development as therapeutics for critical diseases. Alzheimer’s disease is a progressive and evolving neurodegenerative disease with an insidious onset, and its etiology remains unclear to date. To investigate the effects of flavonoids on neuroinflammation associated with Alzheimer’s disease, Zhang et al. reviewed the relevant pharmacological effects and mechanisms of 13 natural flavonoids (apigenin, luteolin, naringin, quercetin, morin, kaempferol, fisetin, isoquercetin, astragalin, rutin, icariin, mangiferin, and anthocyanidin) extracted from plants or medicinal herbs [177]. Gan et al. summarized and discussed the origin, refined extraction, biosynthesis, metabolism, and bioactivity of polymethoxyflavones (PMFs), an uncommon dietary flavonoid present in plants such as *Citrus reticulata* and *Kaempferia galanga*, focusing on the molecular mechanisms by which PMFs modulate different chronic diseases [178]. Kaempferol and its derivatives have been shown to have antioxidative stress, blood–brain barrier protection, and neuroprotective effects via preventing the deposition of amyloidogenic fibrils (such as aβ, τ, and α-synuclein), inhibiting microglia activation, reducing the release of inflammatory factors, and restoring the structure of mitochondrial membranes [179]. Quercetin mitigated corticosterone (CORT)-induced depression-like behaviors and the mechanism was partly related to the repression of neuroinflammation and oxidative damage [180]. Because proanthocyanidins have strong antioxidant properties, they can effectively reduce the symptoms of skin allergies and allergic asthma by blocking the production and release of inflammatory factors like histamine, 5-hydroxytryptamine, prostaglandins, and leukotrienes as well as the release of allergic particles from basophils and mast cells [181]. Furthermore, it has the potential to greatly alleviate the symptoms of a number of illnesses, such as gastrointestinal and arthritis, by blocking the actions of hyaluronidase and histamine decarboxylase [182,183]. It has been recorded that proanthocyanidins have improved anti-radiation effects [184].

Furthermore, some flavonoids, such as isoflavonoids from whole grains, nuts, soybeans, and flaxseeds, can change the levels of hormones in the body. In high estrogen environments, they can act as estrogen antagonists, and in low estrogen environments, they can act as estrogen agonists [185]. The development of depression is accompanied by changes in the type and number of intestinal microorganisms, flavonoids can modulate the type and structure of intestinal microorganisms, and gut microbes can significantly improve depressive symptoms through the gut–brain axis [186]. Licoflavone B is a flavonoid isolated from *Glycyrrhiza inflata* which showed significant anti-schistosomal effects. The IC_50_ values were 23.78 µM and 31.50 µM for the inhibition of ATPase and ADPase of *Schistosoma mansoni*, respectively [187]. Research on the pharmacological actions of flavonoids will therefore increase in tandem with the rise in new ailments.

## 5. Pharmacological Targets and Mechanisms of Action of Flavonoids

### 5.1. Mechanisms of Antioxidant 

Hydrogen atoms are transferred to free radicals by flavonoids as part of their antioxidant process. The higher the flavonoid’s antioxidant capacity, the faster and easier the hydrogen transfer is made by the flavonoid structure. As a result, among the various flavonoids, the ones with hydroxyl groups in their structure have the most antioxidant activity [188]. There are two ways in which flavonoids influence the intricate biological process of lipid peroxidation: (I) Free radicals are directly scavenged by flavonoids. They have the ability to stop the spread of free radicals and halt the chain reaction that is brought on by a variety of physicochemical variables. Additionally, flavonoids can lessen biofilm damage by stopping unsaturated fatty acids and arachidonic acids from peroxidizing. Furthermore, flavonoids can also directly eliminate singlet oxygen and hydroxyl radicals by single electron transfer. (II) Flavonoids have indirect scavenging effects on free radicals in the body. They can coprecipitate with proteins, thus acting on enzymes related to free radicals such as quercetin which can inhibit the activity of xanthine oxidase and quercetin and morin which have an inhibitory effect on cytochrome P450. Flavonoids can also complex metal ions with induced oxidation, inhibiting a variety of oxidation processes involving such metal ions in vivo. For instance, the antioxidant activity of quercetin and rutin in the oxidative system involving Fe^2+^ is related to their ability to complex Fe^2+^. Furthermore, flavonoids and specific chemicals have a synergistic impact that can greatly boost the antioxidant activity of the former. For instance, combining catechins with Vitamins C and E improves their antioxidant activity.

### 5.2. Mechanisms of Anti-Inflammatory 

**Figure 8:**
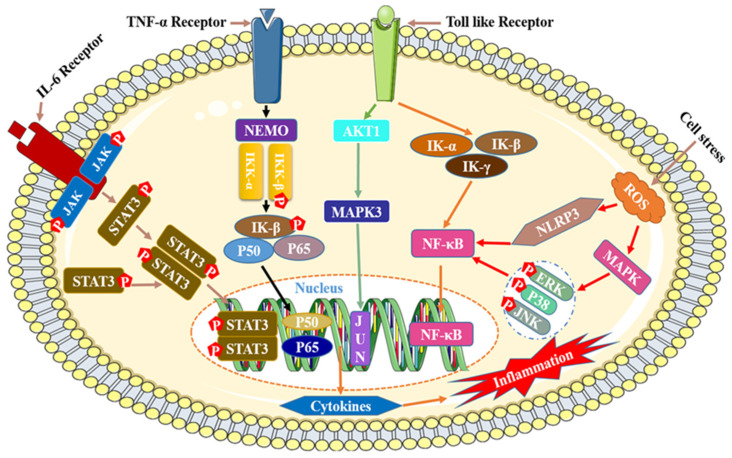
Mechanisms of anti-inflammatory effects of flavonoids.

The mechanism of inflammation is relatively complicated, and currently there are four main reported mechanisms of inflammation, namely IL-6/STAT3,TNF-α/NEMO/IκB, Toll/NF-κB, and Ros/MAPK/NLRP3/NF-κB (Figure 8) [189,190]. 

Although the process by which flavonoids reduce inflammation is quite intricate, it can be summed up as follows: by influencing the expression of significant activated proteins and transcription genes in the signaling pathway, flavonoids block the activation cascade of the signaling pathway, which in turn reduces the secretion and release of inflammatory substances, inflammatory proteins, and inflammatory factors while also having an anti-inflammatory effect. The following four categories best capture the mode of action of flavonoids that have anti-inflammatory effects: (I) preventing the p50 and p65 subunit nuclear translocation and phosphorylation of IκB protein, which lowers the activity of the NF-κB signaling pathway and inactivates inflammatory factors [191,192,193]; (II) blocking the phosphorylation of the activated proteases JNK/stress-activated protein kinase (SAPK), ERK1/2, and p38MAPK, or inhibiting the transcription of the anti-lipolytic genes perilipin and PDE3B, regulating the expression of the MAPK family signaling pathway [194,195,196,197]; (III) blocking the phosphorylation of the upstream kinase JAK2 and decreasing the secretion of iNOS and NO in macrophages [198,199]; (IV) decreasing the activity of signaling pathways and inflammatory factors/proteins by decreasing or blocking the activity of NF-κB, MAPK/ERK, signal transducer, activator of transcription 1 (STAT-1), etc. [200].

In addition, flavonoids also have inhibitory effects on the PI3K/Akt, MAPK, and vascular endothelial growth factor (VEGF) signaling pathways, which play important roles in regulating cell proliferation, cell survival, cell differentiation, cell migration, promoting angiogenesis, and tumor growth [160,201]. Shao et al. demonstrated that *Artemisia anomala* extract may exert its anti-inflammatory effects by activating the TLR4-MyD88-NF-κB signaling pathway, which inhibits phagocytosis, NO content, and the levels of the proinflammatory cytokines TNF-α, IL-6, and IL-10 [202]. 

### 5.3. Anti-Cancer Mechanisms

Apigenin could promote autophagy via upregulating Beclin 1, ULK1, ATG5, ATG13, and LC3B and downregulating AMPK, mammalian target of rapamycin (mTOR), P70S6K, and ATG4. Furthermore, apigenin could inhibit tumor tissue proliferation and restrict tumor growth via ferroptosis in vivo [203]. Zhang et al. showed that flavonoids such as chrysin could cause human esophageal squamous carcinoma KYSE-510 cells to stagnate in the G2/M phase and ultimately cause apoptosis through the upregulation of p21, p53 (p53-inducible gene 3, PIG3), and the downregulation of cyclin B1 [204]. In human gastric adenocarcinoma SGC7901 cells and colon cancer SW480 cells, quercetin can dramatically trigger apoptosis in a time-dependent manner. Its mode of action is to decrease STAT3 protein phosphorylation and mRNA expression, which in turn causes a decrease in the expression of the survivin protein and mRNA, the apoptosis suppressor gene [205]. In addition, quercetin can inhibit the expression of the B-cell lymphoma-2 (Bc1-2) gene in gastric cancer cells and upregulate the expression of BCL2-associated x (Bax) gene, reduce Bcl-2/Bax, and promote cell apoptosis [206]. Additionally, quercetin can raise the amount of Bax protein in human promyelocytic leukemia HL-60 cells, cause apoptosis, and downregulate the Bcl-2 family proteins [207].

Oroxylin A’s primary anti-tumor mechanisms of action include causing tumor cells to undergo apoptosis, preventing tumor cell invasion and metastasis, influencing the glycolytic process of tumor cells, preventing tumor cell proliferation and vascular growth, reversing drug resistance in tumor cells, stopping the cell cycle, etc. [208]. Kim et al. showed that eupatilin inhibited the growth of human breast epithelial MCF10A-ras cell growth in a dose- and time-dependent manner; the mechanism of action was to inhibit the expression of cyclin D1 and B1, CDK1 and 2, while increasing the expression of p53 and p27. Among these, the decrease in cyclin D1 expression may be regulated by the Raf/MEK/ERK signaling pathway [209]. 

Through the Egr-1-dependent Bax pathway, eupafolin can downregulate the expression of cyclin D1 and upregulate the expression of cyclin B1, p53, and p21. This causes the cell cycle to arrest cervical cancer HeLa cells in the G2/M phase and induces cancer cells to undergo apoptosis [210]. Another study found that luteolin inhibits the growth of MDA-MB-231 estrogen receptor-negative breast cancer cells by decreasing the expression of Akt, polo-like kinase (PLK1), cyclin B1 and A, CDK1 and 2, and Bcl-xL, and by increasing the expression of p21 and Bax, leading to the arrest of the cell cycle in the G2/M and S phases, as well as apoptosis [211]. Furthermore, it has been suggested that kaempferol’s anti-inflammatory and antioxidant qualities may be responsible for its capacity to guard against DNA damage and prevent the growth of cancer cells. Moreover, Wang et al. proved that the regulation of M1-like polarization in macrophages by oroxylin-A-induced extracellular vesicles of cancer cells was achieved by caspase-3-mediated activation of ROCK1 in vitro [212].

### 5.4. Anti-Anxiety Mechanisms

Numerous animal anxiety models have demonstrated the significant impact of dietary phytoconstituents such flavonoids, according to extensive experimental research. The GABAA receptor is involved in the mechanisms of action of anxiolytics on both non-BZD and benzodiazepine (BZD) sites [138]. The mechanism of anxiolytic action of chrysin is that it acts as a competitive ligand for benzodiazepines (BZDs) (Ki of 3 μmol/L), and chrysin exhibited anxiolytic activity when administered intraperitoneally to mice at a dose of 1 mg/kg. Apigenin (3 mg/kg) exhibited anxiolytic activity by binding to the BDZ receptor in the GABA-BDZ-Cl receptor complex and enhancing GABAergic neurotransmission (Table 5) [213]. The binding degree (Ki) of wogonin to benzodiazepine receptors (BDZ-S) was 0.92 μmol/L. Wogonin increased the activation current of aminobutyric acid as detected by electrophysiological techniques, and its anxiolytic effect could be inhibited by Ro15-1788, a BDZ-S antagonist [214]. **6**-Hydroxyflavonoids (**6**HF) were found to increase the activation current of aminobutyric acid through benzodiazepine receptors in rat cortical neurons, and its anxiolytic effect could be antagonized by flumazenil, a GABA receptor antagonist [215], confirming once more that benzodiazepine receptors were linked to its anxiolytic action. According to Aya et al., hesperidin and hesperitin groups were able to decrease the amount of time that mice were immobile in the open field when compared to the normal group, without affecting the mice’s voluntary activity behavior. This suggests that at 50 mg/kg, hesperidin and hesperitin exhibit similar anxiolytic activity to fluoxetine, with hesperitin’s effect being slightly stronger than hesperidin’s. It is hypothesized that the mechanism by which hesperidin induces an anxiolytic effect may be connected to its glycoside ligand, hesperitin [216].

The 15 types of natural flavonoid monomer compounds with anti-anxiety effects have been summarized and analyzed in recent years. Flavonoids, flavonols, and dihydroflavones were the main ones, among which flavonoids were the most diverse. Structure–activity relationship studies showed that **2**′-hydroxyl, **8**-position methoxy, and 6-position substitutions contribute to the anxiolytic activity of flavonoids, which decreases as the number of hydroxyl groups increases. The activity of flavonoid glycosides was significantly stronger than that of the corresponding glycosides. These results clearly have a significant impact on the development and application of flavonoid-rich medicinal plant resources as well as the structural optimization of flavonoid anxiolytic medications. Furthermore, Vissennon et al. boldly predicted that the anxiolytic activity exhibited by flavonoid monomers may be related to their degradation products. This hypothesis’s formulation provides a fresh perspective and method for thoroughly elucidating flavonoids’ anxiolytic mechanism of action.

### 5.5. Mechanisms of Antidiabetic Action

To summarize, flavonoids cause a drop in blood sugar levels by acting on several different mechanisms: (I) increasing insulin secretion; (II) controlling important enzymes involved in glucose metabolism; (III) controlling the expression of proteins linked to the insulin signaling pathway and improving insulin sensitivity; (IV) increasing the uptake of glucose by skeletal muscle and white adipose tissue; (V) reducing oxidative stress and inflammation; (VI) improving lipid metabolism and reducing lipotoxicity; (VII) protecting renal tissues and reducing nephrotoxicity. The regulation of these effects mainly focuses on key enzymes in hepatic glycolysis and gluconeogenesis as well as signaling pathways such as AMPK, peroxisome proliferator-activated receptors γ (PPARγ), and PI3K/Akt in liver and fat. Yang et al. investigated the effect of total flavonoids of *Hippophae rhamnoides* (TFH) on improving the symptoms of a type 2 diabetes model in rats. The results indicate that TFH downregulates expressions of protein kinase C alpha (PRKCA), MAPK10, and p65 TNF-α as well as the level of the key metabolite DA in the DAG/PRKCA/MAPK10/TNF-α/p65 pathways, improves lipid metabolism disorder, inhibits inflammatory response, and thereby relieves symptoms of diabetes mellitus type 2 (T2DM) [223]. 

Apigenin, luteolin, and baicalein significantly inhibited the production of ROS and AGEs, which were closely related to the suppression of the phosphorylation form of NF-κB and P65 [159]. Naringenin upregulated the expression of PPARγ, heat shock protein 72 (HSP-72), and HSP-27 in the livers of diabetic rats, thereby increasing the phosphorylation of IRS1 (Tyr162), which ultimately served to lower blood glucose and alleviate insulin resistance [224]. Puerarin had a hypoglycemic effect via reducing oxidative stress and enhancing mitochondrial performance through the modulation of AMPK [225]. Numerous investigations have demonstrated that puerarin can cause hypoglycemia via increasing the expression of PPARα, insulin-like growth factor-1 (IGF-1), and insulin receptor substrate-1 (IRS-1) [226]. Puerarin also acts through the phosphorylation of GSK-3β, downregulation of uncoupling protein 2 (UCP2) mRNA expression levels, and activation of the AKT pathway downstream of the insulin receptor to lower blood glucose, protect pancreatic β-cells, and enhance liver function, according to another study [227]. Additionally, by upregulating the expression of GLP-1R and pancreatic and duodenal homeobox-1 (PDX-1), puerarin enhances glucagon-like peptide-1 receptor (GLP-1R) signaling. This results in the activation of protein kinase B and the inactivation of FOXO1, which promotes β-cell proliferation and reduces β-cell apoptosis. Ultimately, this helps to improve glucose tolerance and reduce body weight in diabetic mice induced with a high-fat diet [228].

Genistein has the ability to operate directly on pancreatic β-cells, boosting insulin production, mending injured β-cells, and increasing the number of insulin receptors via activating the cAMP/PKA signaling pathway [229]. In the treatment of diabetic rats with biochanin A, it was found that it could control body weight gain, reduce blood lipids, protect the pancreas, as well as reduce the activities of AST, ALT, and ALP back to normal. Isoflavonoids have been reported as maintaining glucose homeostasis in vivo mainly by protecting the mass and function of β-cells, which cannot be separated from their modulation of insulin signaling pathways and related molecular targets in the liver and adipocytes. One of them is biochanin A, which, in order to combat oxidative stress and lipid metabolism disorders, specifically improves glucose intake by upregulating visfatin expression. This is crucial for the treatment of diabetes mellitus caused by obesity. Epigallocatechin gallate (EGCG) can directly act on NO synthase in skeletal muscle to have a vasodilatory effect. It also has an insulin-mimetic effect on glucose metabolism [230]. In addition, EGCG can activate AMPK and PGC-1α, which in turn inhibits ACC activity and promotes the expression of GLUT4, thus alleviating insulin resistance [231]. Both luteolin and luteolin-7-O-glucoside could significantly reduce the blood glucose and glucose tolerance, and improve insulin resistance in KK-Ay mice. The mechanism involved lowering TG content and the expression of fatty acid synthase (FAS) and SREBP-1c, inhibiting lipid synthesis, and reducing serum TNF-α levels, exerting anti-inflammatory activity, reducing hepatic thiobarbituric acid reactive substances (TBARS), and resisting oxidative stress [232]. Swertisin can act as an islet differentiation inducer, efficiently promoting the differentiation of pancreatic stem/progenitor cells into pancreatic β-cells, activating p38MAPK, neuregulin-3, Smad protein cascade, and MEPK-TKK signaling pathways, and it is a valuable antidiabetic drug targeting endogenous cell differentiation [233]. The research has indicated that the mechanism by which flavonoids cause hypoglycemia is centered around their capacity to safeguard β-cell activity, initiate signaling pathways in the liver and adipose tissue, and enhance energy metabolism and insulin sensitivity. However, research on the antidiabetic activity of flavonoids is relatively limited in vivo; thus, studies on the activity of flavonoids targeting key targets are the main direction at present in vivo. It has been established that the way in which anthocyanins regulate glucolipid metabolism is through their inhibition of hepatic gluconeogenesis, insulin resistance, lipid accumulation and oxidation, and the enzymatic activities of PCG-1α, PEPCK, and G-6-Pase. Combining these kinds of foods in the diet would be one approach to successfully avoid diabetes and its complications because anthocyanins are present in a variety of foods.

### 5.6. Mechanisms of Analgesic Action

According to studies, rutin can lessen pain brought on by granulocyte colony-stimulating factors by upregulating the expression of the anti-nociceptive allergy cytokine IL-10, activating the NO-cGMP-PKG-KATP pathway, inhibiting the NF-κB pathway, and triggering the Nrf2/HO-1 pathway to prevent the production of IL-1β and TNF-α [234]. Matrix metalloproteinases (MMP) mediate neuroinflammation by cleaving extracellular matrix proteins and activating proinflammatory cytokines. Naringenin reverses neuropathic pain by inhibiting the expression of MMP and decreasing the production of TNF-α and TGF-1β [235]. Hesperidin has anti-inflammatory as well as analgesic effects, and its combination with NSAIDs synergistically can reduce the pain response and decrease the dose of NSAIDs [236]. Furthermore, hesperidin methylation results in hesperidin methyl chalcone (HMC), which has been shown to directly interact with p65’s Ser276 to lessen the activation of the NF-κB pathway in macrophages. This lowers the levels of NF-κB-dependent IL-33, TNF-α, and IL-6 and relieves the reduction in arthritis pain caused by yeast polysaccharides [237]. H_2_O_2_ significantly increased the expression of cGAS, Sting, and NLRP3 protein levels, and EGCG treatment demonstrated significant protective effects in cell viability, apoptosis, cell cycle arrest, and inflammatory status through the downregulation of the cGAS/Sting/NLRP3 pathway [238]. Thus, the primary mechanism of action of flavonoids’ analgesic effect is to prevent the activation of inflammatory pathways and decrease the generation of inflammatory factors.

### 5.7. Mechanisms of Action on Alcoholic Liver Disease

In order to prevent HSC activation and lessen liver fibrosis, wogonin was able to increase the activation of caspase-9 and caspase-3 and raise the ratio of Bax/Bcl-2 [239]. In addition, wogonin has been shown to induce hepatocellular carcinoma cell cycle block, regulate cellular metabolism, and inhibit tumor proliferation by targeting GSK-3β [240]. Oroxylin attenuates hepatocyte pyroptosis by suppressing caspase-1 activation in the NLRP3 inflammasome pathway [241]. Quercetin is thought to alleviate alcoholic fatty liver in mice by inhibiting ethanol-induced overexpression of the lipid droplet-related protein perilipin 2 (PLIN2), increasing autophagic lysosomes to rise, improving lipid autophagy, and restoring mitochondrial architecture [242]. Quercetin could effectively inhibit the lipid hyperaccumulation, inflammatory response, and oxidative stress caused by alcohol ingestion by blocking P2X7R and inhibiting the production of the NLRP3 inflammasome [243]. The hesperidin derivative, **4**-methyl coumarin-[**5**,**6**-g]-hesperidin(**4**-MCH), significantly reduced the release of IL-6 and TNF-α, inhibited the activation of NF-κB, and upregulated the protein expression of PPARγ in alcohol-stimulated primary macrophages in mice and RAW264.7 cells, thereby alleviating the alcohol-induced hepatic inflammatory response [244]. Puerarin was able to attenuate alcohol-induced hepatic steatosis by modulating the AMPKα/ACC signaling pathway [245]. Furthermore, puerarin has been shown to lessen ethanol-induced chronic alcoholic liver injury in rats via modifying the pathways for COX-2 and arachidonic acid-5-lipoxygenase (ALOX5) [246].

### 5.8. Mechanisms of Antiviral Action

Researchers are eager to screen natural materials, particularly flavonoids, for active components that have antiviral action. Studies have shown that a variety of viruses, including adenoviruses, coronaviruses, polioviruses, respiratory syncytial viruses, herpesvirus, coxsackievirus, hepatitis virus, and dengue virus, are inhibited by flavonoids. It has been reported that isoscutel-larein-8-methyl ether can significantly inhibit the influenza virus, and rutin has the effect of inhibiting the influenza virus and poliovirus. Wang et al. found that total flavonoids of Astragalus had a good therapeutic effect on human herpesvirus (HSV21)-infected guinea pig skin [247]. The total flavonoids of Viola kunawarensis var. (TFVK) were tested for their antiviral activity using Western blotting and cytopathic effect (CPE) methods in vitro and in vivo. The results indicated that TFVK had significant anti-influenza viral effects both in vitro and in vivo; the mechanism of action may have been through the direct inhibition of M2 and NS1 protein expression. Furthermore, by blocking the expression of p65 protein and its nuclear translocation in host cells and interfering with the expression of inflammatory proteins downstream of the NF-κB signaling pathway, TFVK may have indirect antiviral effects [248]. A cell-based SARS-CoV-2 replication assay to screen 1019 different flavonoids for drugs with antiviral activity, among which apigenin and the galloylated pinocembrin analog, pinocembrin **7**-O-(**3**″-galloyl-**4**″,**6**″-(S)-hexahydroxydiphenoyl)-beta-D-glucose (PGHG), are the most potent SARS-CoV-2 inhibitors [249]. At the chosen concentrations (12.5 μg/mL), the dichloromethane fraction of Rhus retinorrhoea’s sakuranetin (SEK) and velutin (VEL) effectively suppressed HBsAg by about 58.8 and 56.4%, respectively, and HBeAg by about 55.5 and 52.4%, respectively. Additionally, stable complexes with good docking energies were formed when the flavonoids were molecularly docked with HBV polymerase and capsid proteins, validating the flavonoids’ structure-based antiviral mechanism [250]. Luteolin is effective in inhibiting the replication of viruses such as coronaviruses, influenza viruses, enteroviruses, rotaviruses, herpesviruses, respiratory syncytial viruses, etc. It inhibits several pathways linked to viral infection and replication, including PI3K-AKT, TLR4/8, NF-κB, Nrf-2/HO-1, and MAPK. Additionally, it boosts the body’s antioxidant and non-specific immunity to avoid viral infections. Furthermore, luteolin inhibits viral replication in cells via controlling the expression of several receptors and cytokines, such as p53, NLRP3, TNF-α, hepatocyte nuclear factor 4α (HNF-4α), and interleukins [251].

## 6. New Drug Development and Clinical Synergy

Many novel medications containing flavonoids or the entire flavonoid content of natural products are currently available on the market. The oral patch (Kang-En-Bei), for instance, is primarily used to treat mild recurrent oral ulcers caused by accumulated heat in the heart and spleen. Symptoms of these ulcers include ulceration of the oral mucosa, localized redness and swelling, burning pain, and so forth. The oral patch is made of total flavonoids from the flowers of *Abelmoschus manihot*. The world’s first new Chinese medicine for the prevention and treatment of urolithiasis is a total flavonoid capsule (Trade Name: Guangshitong^®^; Jiulong Renfu Pharmaceutical Co., Ltd, Wuhan, China), which was created from the total flavonoids isolated and purified from *Desmodium styracifolium*. It has the properties of a heat-clearing, dampness-removing, diuretic, and stone-removing medication. The primary ingredients of *Pueraria lobata*, which was a proprietary Chinese medicine, were replaced with total flavonoids. These flavonoids, which were developed from flavonoid compounds such as puerarin, hydroxy puerarin, methoxy puerarin, and daidzin, are now primarily used for the treatment of cardiovascular and cerebrovascular diseases, such as hypertension, hyperlipidemia, migraine, coronary heart disease, myocardial infarction, angina pectoris, retinal artery obstruction, retinal vein obstruction, and sudden deafness, among others. In order to limit mycoplasma pneumoniae infection, apigenin activates PPARγ, enhances Uhrf1 mRNA expression, increases TNF-α DNA methylation of promoter, and reduces TNF-α mRNA expression. This decreases TNF-α autocrine activity and inhibits the necrotic apoptosis of alveolar macrophages [252]. This research has discovered a new mechanism by which apigenin inhibits alveolar macrophage damage, which has important implications for the treatment of mycoplasma pneumonia.

Moreover, in therapeutic application, flavonoids have shown positive synergistic pharmacological benefits. Flavonoids have varying degrees of therapeutic and preventative effects on colorectal cancer (CRC), and they may work in concert with other anti-CRC therapeutic drugs to enhance apoptosis, prevent carcinogenesis, and slow the growth of tumors. Among these, luteolin may work in concert with lysosomal adenoviruses to increase the cytotoxicity and apoptosis of tumor cells while having no effect on other normal lung epithelial cells. It may also lessen the resistance of CRC tumor cells to the CRC chemotherapeutic agent oxaliplatin by influencing the Nrf2 pathway. Furthermore, in CRC patients, EGCG or resveratrol together with oxaliplatin and cisplatin enhanced apoptosis and decreased tumor cell proliferation [253].

Studies have shown that three catechol-type flavonoids, including flavonoids **7**,**8**-dihydroxyflavone, myricetin, and luteolin, are able to convert iron to the ferrous form to disrupt the iron homeostasis of bacteria, thus having a significant synergistic effect with colistin. Furthermore, catechol type flavonoids also increase the effectiveness of colistin by modulating bacterial membrane charge through interfering with the two-component system pmrA/pmrB, which encourages colistin binding to bacteria and exacerbates bacterial membrane damage [254]. In response to the urgent need for new influenza therapeutics, modified baicalin is currently in phase II clinical trials as a therapeutic agent for adult influenza. Baicalin has also shown exciting pharmacological activities in the screening of anti-SARS coronavirus type 2 compounds, and thus has a wide range of development prospects [255]. Moreover, puerarin and naloxone together can enhance the healing outcome of treating traumatic cerebral infarction [256].

## 7. The Focus of Future Research on Flavonoids

### 7.1. Existing Issues

To summarize, flavonoids exhibit a range of pharmacological properties, including anti-inflammatory, antioxidant, antidiabetic, antitumor, and antiviral properties [257]. However, their limited application in food and medicine can be attributed to their poor hydrophilicity and lipophilicity, poor chemical stability, and low bioavailability, which are caused by their shared planar-type molecular structure. One of the primary drawbacks of flavonoids in various uses is their limited bioavailability. At the moment, creating nanosystems, enzymatic methylation, microemulsions, and microcapsules made of flavonoids are ways of increasing their bioavailability [258,259]. To solve the problem of poor solubility and bioactivity, Islam et al. [260] developed water-soluble micellar formulations containing single and multiple flavonoids using the biocompatible surface-active ionic liquid choline oleate, and investigated the effect of dosage form change on their food preservation properties using luteolin, naringin, and quercetin as models of bioactive compounds. The results showed that the micellar formulations formed spherical micelles with particle sizes < 150 nm and exhibited high water solubility (> 5.15 mg/mL). Following delivery using LNQ-MF (luteolin, naringenin, and quercetin-loaded micellar formulation), a number of flavonoids, including luteolin, naringin, and quercetin, demonstrated 84.85% antioxidant activity at a concentration of 100 mg/mL. In the meantime, LNQ-MF co-delivery had a synergistic impact with partial inhibitory concentration indices of 0.87 and 0.71 μg/mL, respectively, on the anti-Salmonella enteritidis and anti-Staphylococcus aureus of flavonoids. Additionally, encapsulated flavonoids that are co-delivered show promise as a substitute for chemical preservatives. By combining them with varying concentrations of baicalein through an integrated procedure and physical modification, Sun et al. created hydrophobic fibers with antioxidant (baicalein) storage. They also created continuous, beadless, naturally sourced polyvinyl alcohol (PVA)/sodium alginate (SA)-based nanofibers using electro-spinning and physical cross-linking techniques [261]. 

At present, there are several studies on oral delivery systems to improve the stability and bioavailability of quercetin, a representative flavonoid compound, such as quercetin nanomicelles [262] and quercetin nanoparticles [263]. Although there are still issues, these delivery methods give quercetin some initial protection and somewhat increase its stability and bioavailability. As an illustration, (I) quercetin and the conventional oral delivery method are mostly bonded by non-covalent interactions including hydrogen bonds and charge interactions [264]; however, because the non-covalent connection is vulnerable to the ions and acidic environment of the gastrointestinal track, the system’s stability is not optimal. (II) It is challenging to achieve effective intestine-targeted delivery because the majority of conventional carriers are not acid-resistant and have low stability in gastrointestinal fluids with a short retention time. (III) It has been demonstrated that a significant amount of reactive oxygen species (ROS) are released during intestinal inflammation. These ROS have the potential to oxidize quercetin into quinone, rendering it inactive. Therefore, it is unclear if quercetin remains active after being released in the intestine. It is therefore imperative to develop a quercetin delivery system that is acid-resistant, antioxidant-type, and has a high intestine targeting effectiveness. It has been reported that sulfhydrylated carriers can achieve intestinal adhesion by forming disulfide bonds with sulfhydryl groups on mucins in intestinal mucus [265], and selenium-containing compounds can increase glutathione peroxidase (GPx) levels in the body, preventing the accumulation of free radicals, and thus functioning as ROS scavengers [266]. Konjac glucomannan (KGM) is a class of water-soluble polysaccharides extracted from the tuberous roots of the Amorphophallus, with antioxidant, immunity-enhancing, improving blood glucose, blood lipid levels, and other effects, and it is the most viscous class of plant polysaccharides known to date [267]. Because of its degradability in vivo and good biocompatibility, KGM has been increasingly used as a drug carrier in recent years [268]. One innovative method for the effective intestinal-targeted delivery of quercetin and other flavonoids is the use of an acid-resistant, ROS-scavenging oral system for quercetin-targeted delivery. This system is made with calcium lactate as the cross-linking agent, selenated KGM (SeKGM) and thiolated sodium alginate (TSA) as raw materials. Due to their widespread use in medicine and extensive research, flavonoids have been shown to have certain drawbacks, including the inability to accurately determine their content and the potential for harmful side effects from clinical application, including pyrogenic reactions, allergic reactions, shock, and even death. Research has indicated that the creation of charge complexes with a certain kind of lipoprotein may be the origin of the harmful effects of flavonoids. Because flavonoids have a cross-conjugated structure, electron transfer and rearrangement can produce oxonium and carbon-positive ions, which can give rise to both positive and negative charges in them as well as double-pair charge complexes with the positive and negative charges of proteins. The primary cause of the harmful side effects of flavonoids in clinical settings, particularly those resulting from injections of flavonoids, is the strong binding of this charge complex, which makes it challenging to extract using conventional separation techniques.

Furthermore, plants have a lengthy growth cycle and a generally low flavonoid content, making them vulnerable to constraints imposed by time, geography, environment, and a host of other variables. When synthesizing natural goods, chemical synthesis methods still have significant environmental contamination hazards, high costs, complex operations, and low efficiency. Even though many flavonoids have been synthesized in microorganisms with success, low production efficiency remains a challenge in the manufacture of many flavonoids. The primary techniques involve controlling the flow of carbon sources, modulating co-cultivation, and manipulating gene expression to enhance the output of flavonoids; however, the yield increase is insufficient. In conclusion, more study and development are still needed to produce flavonoids with the right bioavailability and pharmacological action.

### 7.2. Future Prospects

Research on the pharmacological activities of flavonoids is currently at a flourishing stage. Numerous flavonoids’ pharmacological activities and mechanisms of action are constantly being clarified, and this research will lead to new and major advancements in the field of flavonoid research. Future research will primarily focus on three areas: the development of novel drug delivery vehicles, the functional investigation of key genes in the flavonoid metabolic pathway, and the change in the chemical structure of flavonoid molecules. First of all, the chemical structure modification of flavonoid compounds is one of the important methods for new drug development [269]. Among them, the synthesis or modification of flavonoids’ C ring and the selective insertion of functional groups onto the A and B rings are crucial methods for producing compounds with strong biological activity and are also the focus of intense research in the field of flavonoid chemical structure modification. Secondly, the resolution of biological metabolic pathways and the development of cellular synthesis techniques are important means of addressing the problem of limited material sources of flavonoid compounds [66]. The simplification of the design and engineering construction of metabolic pathways will be greatly aided by accelerating the innovation of metabolomics and genomics research methods and technologies, continuously enhancing the efficacy of identifying flavonoid-producing gene clusters, and modularizing key gene elements for flavonoid-production into standard “production parts” through protein-directed evolution, metabolic mutations, gene editing, etc. Thus, the key to making progress in future research is to reduce non-target metabolic pathways and simplify the genome of chassis cells.

In addition, the antimicrobial effects of flavonoids have been the focus of research in recent decades [270]. Developing oral dosage forms of flavonoids is made much more challenging by the fact that flavonoids are often poorly soluble in water and are quickly broken down and metabolized by the body. These factors led to the development of nano-formulations and different delivery techniques that improve flavonoid solubility. Among these, encapsulating flavonoids in nanoformulations significantly enhances their pharmacokinetics and safety, whereas dermal administration permits flavonoids to cross the skin barrier to lessen their negative effects. Because of their superior encapsulation ability and low toxicity, flavonoids can be effectively delivered to the skin by integrating them into nanomedicines. The use of nanoparticles or nanoformulations promotes drug delivery by targeting specific action sites and exhibits excellent physicochemical stability [271]. Therefore, the development of new delivery systems will remain the focus of future research on flavonoids.

## 8. Conclusions

Flavonoids are a class of molecules that are commonly found in nature and have a broad variety of pharmacological activities. They are significant therapeutic prospects for the treatment of several infectious and non-communicable diseases. There are fourteen types for flavonoids, most of which come from biosynthesis, chemical synthesis modification, and plant extraction. While the synthetic synthesis of chemical structures plays a significant role in the development of novel therapeutics, biosynthesis is regarded as the most promising area of study and has the ability to completely transform the pipeline for the synthesis of flavonoids under new production restrictions. The creation of an intrinsic link between the pharmacological activity and mechanism of action of flavonoids with the aid of technologies like network pharmacology, metabolomics, proteomics, and genomics can effectively and deeply lay a scientific foundation for the exploitation of the medicinal resources rich in flavonoids. This link can be established with the advancement and development of research technologies across various disciplines.

## Figures and Tables

**Table 1:** Classification and chemical structure of naturally derived flavonoid compounds.

Classification	Skeletons Structure	Representative Compound
Flavones	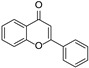	Apigenin, luteolin
Flavonol	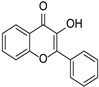	Rutin, quercetin
Flavanones	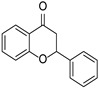	Liquiritin, hesperetin
Flavanonols	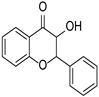	Silybin
Flavan-**3**-ols	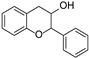	Catechin
Flavan-**3**,**4**-diols	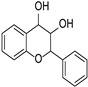	Colorless delphinidin
Isoflavones	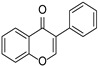	Puerarin, soy isoflavones
Isoflavonones	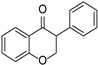	Rotenone
Chalcones	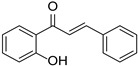	Aureusidin
Aurones	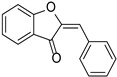	Isoliquiritigenin
Anthocyanidins	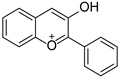	Cyanidin
Biflavonoids	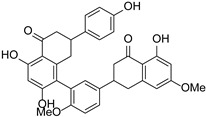	Ginkgetin
Xanthones	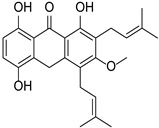	Isomangiferin
Homoisoflavones	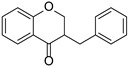	Brazilin

**Table 5:** Mechanism of anti-anxiety effects of flavonoids.

Category	Compound	Mechanism	References
Flavonoid	Chrysin	As a competitive ligand for benzodiazepine receptors, they enhance the GABAergic neurotransmission by binding to the benzodiazepine drug-receptor of the γ-GABA-benzodiazepine-chloride ion receptor complex	[140]
	Apigenin		[213]
	Apigenin-**7**-glucose	Acts on GABA receptors	[217]
	Wogonin	Acts on benzodiazepine receptors for GABA receptors	[214]
	Baicalein	Acts on non-benzodiazepine receptors for GABA receptors	[218,219]
	Baicalin	Acts on non-benzodiazepine receptors for GABA receptors, inhibits the release of glutamate	[219]
	Luteolin	Acts on benzodiazepine receptors	[220]
	**6**-hydroxy flavone	Acts on benzodiazepines receptors for GABAA-type receptors, enhances GABA activation current	[215]
	Amentoflavone	Related to ionic GABA receptors	[221]
	Spinosin	Related to GABA receptors and serotonin receptors	[222]
Flavonols	Kaempferol	Related to GABAA receptors	[141]
	Quercetin		[142]
	Myricetin		[143]
Dihydroflavonoids	Hesperidin	Associated with the serotonin neurotransmitter pathway, acting through its glycosidic ligand hesperidin	[216]
	Hesperetin	Associated with the serotonin neurotransmitter pathway	[216]

## Data Availability

Not applicable.

## Abbreviations

FDA	Food and Drug Administration
UAE	Ultrasound-assisted extraction
RSM	Response Surface Methodology
HPLC	High-performance liquid chromatography
LC-MS	Liquid chromatography tandem mass spectrometry
DES	Deep eutectic solvent
AFO	Algar–Flynn–Oyamada method
BKVK	Baker–Venkataraman method
PTZ	Pentylenetetrazol
NRG	Neuregulin
CDK	Cyclin-dependent kinase
IC_50_	Half maximal inhibitory concentration
COX-2	Cyclooxygenase-2
ITS	Internal Transcribed Spacer
CHS	Chalcone synthase
FNS	Flavonol synthase
CHI	Chalcone isomerase
IFS	Isoflavone synthase
F3′H	Flavonoid 3′-hydroxylase
PAL	Phenylalanine ammonia-lyase
TAL	Tyrosine ammonia-lyase
C4H	Cinnamic acid-4-hydroxylase
4CL	4-coumaroyl-CoA
PEP	Precursors 2-(phenoloxy) prop-2-en-1-olate
E4P	(S)-2-hydroxy-4-oxobutyl dihydrogen phosphate
TcCGT	Trollius chinensis C-glycosyltransferase
GmSUS	Glycine max sucrose synthase
DPPH	2-diphcnyl-l-picrylhydrazyl
ABTS	2,2′-azino-bis(3-ethylbenzothiazoline-6-sulfonic acid)
ROS	Reactive oxygen species
EE	Ethanolic extract
FG	Flavonoid glycosides
NF-κB	Nuclear factor-kappa B
5-HT	5-hydroxytryptamine
PGE2	Prostaglandin E2
HO-1	Heme oxygenase 1
Nrf2	Nuclear factor erythroid 2-related factor 2
LTB4	Leukotrienes B4
TNF-α	Tumor necrosis factor-alpha
IL-6	Interleukin-6
IL-8	Interleukin-8
TLR4	Toll-like receptors 4
Akt	Protein kinase B
NQO1	NAD(P)H: quinone oxidoreductase 1
MyD88	Myeloid differentiation primary response 88
p38MAPK	Phospho-p38 mitogen-activated protein kinases
JNK	c-Jun N-terminal kinase; 6-MeOF, 6-methoxyflavone
LDH	Lactate dehydrogenase
CK-MB	Creatine kinase muscle/brain
CABG	Coronary artery bypass grafting
CVD	Cardiovascular disease
PEPCK	Phosphoenolpyruvate carboxykinase
G-6-Pase	Glucose-6-phosphatase
ACC	Acetyl coenzyme A carboxylesterase
IRS-2	Insulin receptor substrate-2
GLUT4	Glucose transporter protein 4
IR	Insulin-resistant
PI3K	Phosphatidylinositol 3-kinase
IRS-1	Insulin receptor substrate-1
GSK-3β	Phosphoglycogen synthase kinase
AGEs	advanced glycosylation end products
NMR	Nuclear magnetic resonance spectroscopy
MIC	Minimum inhibitory concentration
TC	Total cholesterol
TG	Triacylglycerol
TBIL	total bilirubin
NAFLD	Non-alcoholic fatty liver disease
A2Ar	Adenosine A2a receptor
ERK	Extracellular regulated protein kinases
MMP-2	Matrix Metallopeptidase 2
TGF-β1	Tubuloglomerular feedback β1
BLM	Bleomycin
LC3	Microtubule-associated protein1 light chain 3
PINK1	PTEN-induced putative kinase 1
LAMP1	Lysosome-associated membrane proteins 1
TFEB	Transcription factor EB
EMT	Epithelial–mesenchymal transition
SAPK	Stress-activated protein kinase
STAT-1	Signal transducer and activator of transcription 1
SAPK	Stress-activated protein kinase; mTOR, mammalian target of rapamycin
VEGF	Vascular endothelial growth factor
Bcl-2	B-cell lymphoma-2
Bax	BCL2-associated X
PLK1	Polo-like kinase
PPARγ	Peroxisome proliferator-activated receptors γ
TFH	Hippophae rhamnoides
PRKCA	Protein kinase C alpha
IGF-1	Insulin-like growth factor-1
UCP2	uncoupling protein 2
GLP-1R	Glucagon-like peptide-1 receptor
EGCG	Epigallocatechin gallate
TBARS	Thiobarbituric acid reactive substances
FAS	fatty acid synthase; KGM, Konjac glucomannan
GPx	Glutathione peroxidase
TSA	Thiolated sodium alginate
GPx	Glutathione peroxidase
PVA	Polyvinyl alcohol
SA	Sodium alginate
ALOX5	Arachidonic acid-5-lipoxygenase
MMP	Matrix metalloproteinases
LNQ-MF	Luteolin, Naringenin and Quercetin-loaded Micellar Formulation
T2DM	diabetes mellitus type 2
Sirt3	recombinant Sirtuin 3
YAP1	Yes-associated protein 1
HMC	Hesperidin methyl chalcone
